# Fluoride export is required for the competitive fitness of pathogenic microorganisms in dental biofilm models

**DOI:** 10.1128/mbio.00184-24

**Published:** 2024-04-16

**Authors:** Aditya Banerjee, Chia-Yu Kang, Minjun An, B. Ben Koff, Sham Sunder, Anuj Kumar, Livia M. A. Tenuta, Randy B. Stockbridge

**Affiliations:** 1 Department of Molecular, Cellular, and Developmental Biology, University of Michigan, Ann Arbor, Michigan, USA; 2 Program in Biophysics, University of Michigan, Ann Arbor, Michigan, USA; 3 School of Dentistry, University of Michigan, Ann Arbor, Michigan, USA; Case Western Reserve University School of Medicine, Cleveland, Ohio, USA

**Keywords:** biofilms, dental, transporter, fluoride, caries, CLC, crcB, Fluc

## Abstract

Microorganisms resist fluoride toxicity using fluoride export proteins from one of several different molecular families. Cariogenic species *Streptococcus mutans* and *Candida albicans* extrude intracellular fluoride using a CLC^F^ F^-^/H^+^ antiporter and FEX fluoride channel, respectively, whereas oral commensal eubacteria, such as *Streptococcus gordonii,* export fluoride using a Fluc fluoride channel. In this work, we examine how genetic knockout of fluoride export impacts pathogen fitness in single-species and three-species dental biofilm models. For biofilms generated using *S. mutans* with the genetic knockout of the CLC^F^ transporter, exposure to low fluoride concentrations decreased *S. mutans* counts, synergistically reduced the populations of *C. albicans*, increased the relative proportion of oral commensal *S. gordonii*, and reduced properties associated with biofilm pathogenicity, including acid production and hydroxyapatite dissolution. Biofilms prepared with *C. albicans* with genetic knockout of the FEX channel also exhibited reduced fitness in the presence of fluoride but to a lesser degree. Imaging studies indicate that *S. mutans* is highly sensitive to fluoride, with the knockout strain undergoing complete lysis when exposed to low fluoride for a moderate amount of time. Biochemical purification of the *S. mutans* CLC^F^ transporter and functional reconstitution establishes that the functional protein is a dimer encoded by a single gene. Together, these findings suggest that fluoride export by oral pathogens can be targeted by specific inhibitors to restore biofilm symbiosis in dental biofilms and that *S. mutans* is especially susceptible to fluoride toxicity.

**IMPORTANCE:**

Dental caries is a globally prevalent condition that occurs when pathogenic species, including *Streptococcus mutans* and *Candida albicans*, outcompete beneficial species, such as *Streptococcus gordonii*, in the dental biofilm. Fluoride is routinely used in oral hygiene to prevent dental caries. Fluoride also has antimicrobial properties, although most microbes possess fluoride exporters to resist its toxicity. This work shows that sensitization of cariogenic species *S. mutans* and *C. albicans* to fluoride by genetic knockout of fluoride exporters alters the microbial composition and pathogenic properties of dental biofilms. These results suggest that the development of drugs that inhibit fluoride exporters could potentiate the anticaries effect of fluoride in over-the-counter products like toothpaste and mouth rinses. This is a novel strategy to treat dental caries.

## INTRODUCTION

Dental caries is a globally prevalent disease that affects almost 2.4 billion adults and 621 million children (1, 2). In addition to causing pain and stress, infections resulting from tooth decay can cause 0.1% fatality among patients (3). Dental caries is caused by a pathogenic dental biofilm composed of acidogenic and aciduric species like *Streptococcus mutans* and *Candida albicans*, which feed on a diet rich in fermentable carbohydrates (4). *S. mutans* and *C. albicans* are symbionts, forming mixed-species biofilms that are fitter and more cariogenic than single-species biofilms (5–7). Under sugar exposure, these cariogenic species reduce the biological diversity of the dental biofilm and outcompete oral eubiota like the oral commensal streptococci *Streptococcus gordonii*, *Streptococcus sanguinis*, and *Streptococcus oralis* (8).

Fluoride is a widely accepted anticaries agent present in toothpaste and other dental healthcare products that promotes tooth remineralization (9). Fluoride also exhibits antimicrobial activity due to its broad-spectrum inhibition of essential metabolic enzymes, including enolase in the glycolytic pathway, pyrophosphatase, and ATP-consuming enzymes (10). Over-the-counter fluoride products like toothpaste and rinses contain fluoride in the range of 12 to 60 mM fluoride (226 to 1100 ppm F^-^), which is high enough to have such antimicrobial effects (11). However, during the first hour after using these treatments, fluoride concentrations in the oral fluids drop to <1 mM (12–15). Because most microbes possess fluoride export proteins to maintain cytoplasmic fluoride at low levels (16), the antimicrobial effect is clinically irrelevant at these concentrations. Genetic knockout of fluoride exporters causes fluoride hypersensitivity in multiple species, including oral microbiota, reducing the inhibitory concentration of fluoride to the tens-to-hundreds of micromolar range (17–19). This enhanced toxicity suggests that inhibition of fluoride efflux by pathogenic microbes could potentiate the effects of oral fluoride and reduce dental dysbiosis at clinically relevant fluoride concentrations and exposure times.

**Fig 1:**
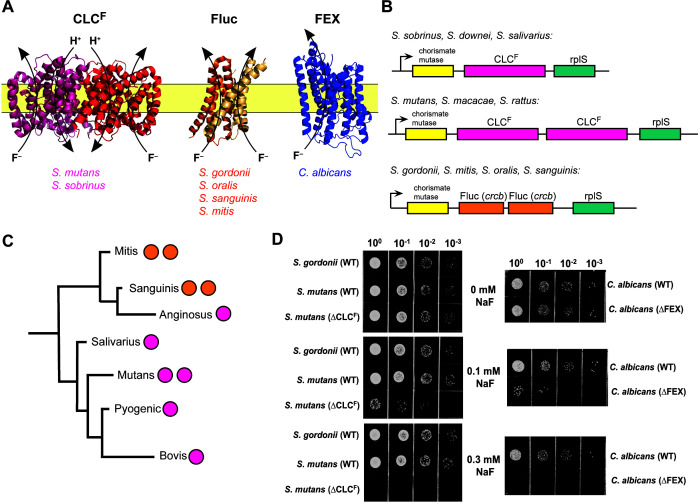
Fluoride exporters from representative oral microorganisms. (A) Structures of fluoride exporters: F^−^/H^+^ antiporter CLC^F^ [PDB: 6D0J (26)]; F^−^ channel Fluc [PDB: 5NKQ (27)]; and F^−^ channel FEX (AlphaFold model). Representative oral species that possess each type of exporter are indicated. (B) Operon architecture of selected oral streptococci. (C) Phylogenetic tree of major streptococcus groups with the number and kind of fluoride exporter genes indicated (CLC^F^: magenta; Fluc: orange). Phylogenetic relationship and branch lengths from reference (28). (D) 10-fold serial dilutions of *S. mutans*, *S. gordonii,* and *C. albicans* on plates containing NaF at the indicated concentration.

Two additional factors suggest that it might be feasible to specifically target fluoride efflux by oral pathogens. First, microbial fluoride sensitivity is amplified by low pH, as is characteristic of dysbiotic dental biofilms (20). This is because fluoride is a weak acid (pK_a_ of 3.4), and its conjugate acid, HF, can readily cross the cell membrane, where it dissociates into H^+^ and F^-^ in the relatively higher pH of the cytoplasm (21). In the absence of a fluoride export mechanism, these impermeable ions become trapped and accumulate intracellularly (20). Second, pathogenic and health-associated oral species possess fluoride export proteins from different molecular families (Fig. 1A). *S. mutans* possesses two genes that encode F^-^/H^+^ antiporters from the CLC (**c**h**l**oride **c**hannel) family of anion channels and transporters, termed CLC^F^ (22). By contrast, oral eubiota including *S. gordonii, S. oralis*, and *S. sanguinis* all export F^-^ *via* the action of Fluc (**Flu**oride **c**hannel) proteins (also known by their gene name, *crcB*), which harness the electrical component of the proton motive force to expel cytoplasmic fluoride (23). Eukaryotes, including *C. albicans*, express a third type of fluoride exporter called FEX (**F**luoride **Ex**porter) (18). These channels are structurally related to the Flucs but possess a more complicated two-domain fold (24, 25).

In this work, we test our hypothesis that the elimination of fluoride export in cariogenic *S. mutans* and *C. albicans* reduces their competitive fitness in mixed-species biofilms, with a concomitant reduction in cariogenic biofilm properties. We examine the species composition and pathogenic potential of dental biofilms composed of *S. mutans*, *C. albicans,* and the beneficial oral commensal *S. gordonii*. We find that genetic knockout of fluoride exporters in the pathogenic species yields biofilms with a larger proportion of oral commensal bacteria, lower total biomass, and reduced pathogenicity at fluoride concentrations as low as 0.2 mM (3.8 ppm F^−^). We further show that *S. mutans* is especially sensitive to the ablation of fluoride efflux, as the CLC^F^ knockout strain exhibits complete lysis upon exposure to 1 mM fluoride for moderate lengths of time. Biochemical and genetic analyses of the two CLC^F^-encoding genes in *S. mutans* show that only one of the two open reading frames contributes to fluoride export by this pathogen. Together, our data indicate that fluoride exporters are potential targets for inhibition to eliminate cariogenic bacteria, reduce dental biofilm dysbiosis, and improve oral health.

## MATERIALS AND METHODS

### Microbial strains

Experiments were performed with *S. mutans* UA159 (ATCC 700610), *S. gordonii* V288 (ATCC 35105), and *C. albicans* BWP17 (29). For *S. mutans,* the SMU.1289 and SMU.1290 open reading frames were deleted by homologous recombination. For the double knockout strain, SMU.1290 and SMU.1289 were deleted sequentially (referred to in the text as ΔCLC^F^). Synthetic genes encoding the spectinomycin and erythromycin resistance genes were prepared with sequence homology to the upstream and downstream regions of 1290 and 1289, respectively, using two-step PCR (30). Primers are reported in Table S1. Electrocompetent *S. mutans* cells were electroporated with 1 µg of the disruption construct, incubated for 2 h in TSB supplemented with 1% (wt/vol) glucose at 37°C under 5% CO_2_ for 2 h, and plated on tryptic soy agar containing 1% (wt/vol) glucose and the appropriate antibiotic. Plates were incubated at 37 °C under 5% CO_2_ for 2–6 days until colonies appeared. Mutants were confirmed using PCR analysis. For complementation experiments, Δ1289 *S. mutans* (erythromycin resistant) were transformed with pDL278 encoding ORF 1289. Transformants were selected with 300 µg/L spectinomycin and protein expression was induced with 0.5 mM isopropyl β-D-1-thiogalactopyranoside (IPTG).

For *C. albicans*, a homozygous FEX deletion (ΔΔ*FEX1*, referred to in the text by the shorthand ΔFEX) was prepared by homologous recombination-based gene replacement. Splice overlap-extension PCR was used to generate a cassette with *HIS1* from *C. dubliniensis* flanked by approximately 300 base pairs of sequence from the region immediately upstream and downstream of the targeted fluoride exporter gene (31, 32). The *HIS1* knockout cassette was introduced into *C. albicans* BWP17 (*ura3::imm434*/*ura3::imm434 iro1*/*iro1::imm434 his1::hisG*/*his1::hisG arg4*/*arg4*) by standard methods of lithium acetate-mediated DNA transformation (33). The site of *CdHIS1* integration in selected transformants was verified by PCR. A homozygous deletion mutant was generated by replacement of the second gene copy using a *SAT1* deletion cassette with flanking sequence homology to the targeted fluoride exporter gene. The *SAT1* knockout cassette was generated as described above. Transformants were selected for growth on yeast synthetic dropout medium without histidine and with 100 µg/mL nourseothricin. Cassette integration was again verified by PCR, and retention of the *HIS1* cassette was also verified in this transformant. The homozygous deletion mutant was subsequently archived as a 15% glycerol stock at −80°C.

### Media and growth conditions

Typically, *S. mutans* was cultured in tryptic soy broth (TSB) with 1% glucose (wt/vol) at 5% CO_2_ and 37°C. For ∆1289 and ∆1290 *S*. *mutans*, media was supplemented with 10 µg/mL erythromycin or 300 µg/mL spectinomycin, respectively, and with both antibiotics for the ∆1290∆1289 double knockout strain (∆CLC^F^). *C. albicans* was typically cultured in yeast peptone dextrose (YPD) (Sigma-Aldrich, St. Louis, MO) supplemented with 25 µg/mL uridine aerobically at 30°C.

For dilution assays, overnight cultures were diluted to adjust the OD_600_ to 0.1. Ten-fold serial dilutions were spotted on tryptic soy agar with 1% (wt/vol) glucose (for *S. mutans*) or Sabouraud dextrose agar (for *C. albicans*) containing NaF as indicated in the text. For the *S. mutans* fluoride survival assays, fresh cultures of ∆CLC^F^ or WT *S. mutans* were grown in TSB with 1% (wt/vol) glucose with antibiotic as appropriate to OD_600_ of 0.3 at 37°C and 5% CO_2_. 50 µL of the culture was used to inoculate 5 mL of fresh media containing 0.3 mM or 1 mM NaF. Cultures were incubated at 37°C for the indicated time, at which point 5 µL of the culture was collected and immediately spread onto tryptic soy agar containing 1% (wt/vol) glucose without any NaF. CFUs were counted after 48-h incubation.

### Transmission electron microscopy

∆CLC^F^ and WT *S. mutans* were incubated for 4 h in TSB supplemented with 1% (wt/vol) glucose and NaF. After pelleting, cells were fixed overnight at 4°C in glutaraldehyde [2% (vol/vol) in 0.1 M Sorensen’s buffer, pH 7.4]. Cells were washed multiple times in 0.1 M Sorensen’s buffer and then post-fixed in 1% (wt/vol) osmium tetroxide for 2 h at room temperature. After additional washes with 0.1 M Sorensen’s buffer, cells were immobilized in 1% (wt/vol) agarose. Agarose slices (~0.5 mm) were dehydrated using four 15 min washes with stepwise increases in ethanol from 25% to 100% and washed three times in 100% propylene oxide. The embedding resin was prepared by mixing 30 g Poly/Bed 812 (PolySciences Inc., USA), dodecenylsuccinic anhydride (DDSA), and nadic methyl annhydride (NMA) in a 2:1:1 ratio in propylene oxide. 2% Tris(dimethylaminomethyl)phenol (DMP-30) was added to the resin as an accelerator. Dehydrated agarose slices were infiltrated with resin in three concentration steps (25%, 50%, and 75%), with 16-h room temperature incubation for each. A final incubation was performed at 60°C under vacuum for 48 h in electron microscopy molds. After polymerization, 50 nm sections were obtained using a Leica UC7 ultramicrotome, loaded onto copper grids, stained in 7% (wt/vol) uranyl acetate for 10 min, washed in deionized water for 10 min, and post-stained in Reynold’s lead citrate for 5 min. Imaging was performed on a JEOL 1400-plus transmission electron microscope equipped with an XR401 AMT sCMOS camera.

### Three-species surface biofilm

Polystyrene stalks (0.25-inch diameter and 0.5 inches in length; United States Plastic Corp, USA) were attached to the inner surface of the lid of a 24-well microtiter plate using double-sided tape and sterilized by ethylene oxide. Inoculum [2 × 10^8^, 2 × 10^8^, and 2 × 10^6^ CFU/mL for *S. mutans*, *S. gordonii,* and *C. albicans,* respectively, according to reference (8), in TSB supplemented with 1% (wt/vol) sucrose] was incubated at 5% CO_2_ and 37 °C for 8 h for initial adhesion to the polystyrene stalks. Stalks were rinsed three times for 10 s in saline [0.9% (wt/vol) NaCl] prior to initiating the experiment, which typically used TSB supplemented with 1% (wt/vol) sucrose, and NaF or NaCl. Biofilms were grown for 16 h at 37°C under 5% CO_2_ without antibiotics. To harvest biofilms, the stalks were rinsed in saline, detached from the lid, and immersed in sterile saline solution in microcentrifuge tubes for sonication using a cup horn sonicator. Serial dilutions of the suspension were plated on Sabouraud dextrose agar (for *C. albicans;* aerobic 30°C incubation, 48 h), Mitis Salivarius agar (for *S. mutans*), or TSB with 1% (wt/vol) glucose (for *S. mutans* and *S. gordonii,* at 5% CO_2_, 37°C incubation, 24 h). To determine biofilm dry weight, the biofilm suspension was pelleted and dried at 37 °C for 3 days.

### Biofilm growth on hydroxyapatite discs

Hydroxyapatite (HA) discs, each 0.5-inch diameter and 0.08-inch width (Clarkson Chromatography Products Inc, USA) were affixed in a vertical position to the lid of a 24-well microtiter plate using double-sided tape and sterilized using ethylene oxide as described (34). Biofilms were formed and adhered as in the polystyrene stalk experiments. After initial adhesion, the discs were washed and immersed in fresh TSB containing 0.1 mM glucose without antibiotic, supplemented with 0.2 mM NaF or NaCl, and incubated at 37°C under 5% CO_2_ for 16 h (famine period). The next day, the discs were rinsed in saline solution and transferred to TSB supplemented with 1% (wt/vol) sucrose (56 mM) supplemented with 0.2 mM NaF or NaCl and incubated for 8 h (feast period). After 5 days of feast-and-famine cycles, the HA discs were rinsed, detached from the lid, and sonicated to detach the biofilm. The suspension was serially diluted onto a selective media described above. The pH of the media was assessed after each media exchange using a calibrated pH electrode. Media calcium content was determined using the colorimetric Arsenazo III reagent (35). Absorbance (650 nm) was measured using a microplate reader (SpectraMax iD3, Molecular Devices, San Jose, CA), and calcium concentration was determined from a standard curve. For fluoride determination, 0.1 mL of 0.5 N HCl was added per 10 mg biofilm wet weight and extracted for 3 h with agitation. Debris were pelleted, and the supernatant was neutralized with TISAB II buffer containing 0.5 N NaOH. Fluoride was measured using a fluoride electrode (96–09, Thermo Fisher Scientific, Waltham, MA), and the concentration was determined from a standard curve prepared using the same reagents as the samples.

### Quantitative PCR

After initial adhesion of the biofilm (WT strains of *S. mutans*, *S. gordonii,* and *C. albicans*), polystyrene stalks were rinsed and re-incubated in 2 mL of fresh TSB containing 1% (wt/vol) sucrose and 0.2 mM NaF or NaCl. Biofilm was harvested after 2, 4, 16, or 24 h of incubation and washed three times in saline with sonication to remove EPS (36). Total RNA was isolated from the biofilm using the TRIzol Max Bacterial RNA isolation kit (Thermofisher Scientific, USA) following the manufacturer’s protocol. RNA (5 µg) was treated with DNaseI (1 U/µL) to remove genomic DNA contamination, then reverse transcribed using Maxima First Strand cDNA synthesis kit (Thermo Fisher Scientific) following the manufacturer’s protocol. For qPCR, gene-specific primers (Table S1) and 10 ng of diluted cDNA template were used. The reaction was performed with Power SYBR Green PCR Master Mix (Thermo Fisher Scientific) in qPCR Step-One Plus detection system (Applied Biosystems, Waltham, MA) using comparative C_T_ method with endogenous control *gyrase A* used to normalize the expression variance of target genes among samples using the 2^-∆∆C^_T_ formula (37).

### Protein purification and proteoliposome preparation

For overexpression and biochemical assays, the coding sequences of CLC^F^ ORF 1289 or ORF 1290 were separately cloned into a pASK vector with a C-terminal hexahistidine tag (22) and transformed into *Escherichia coli* (BL21-DE3). Protein expression was induced at OD_600_ 0.5 with 0.5 mg/mL anhydrotetracycline for 3 h. After cell disruption by sonication, the protein was extracted with 2% (wt/vol) decyl-β-D-maltopyranoside (DM; Anatrace, Maumee, OH) for 2 h at room temperature. The lysate was centrifuged to pellet cell debris, and the protein was purified using cobalt affinity resin (1 mL/L culture, Takara Bio USA, San Jose, CA). The protein was washed with 100 mM NaCl, 20 mM imidazole, 20 mM Tris-HCl pH 8.0, 5 mM DM, and eluted in the same buffer with 400 mM imidazole, before a final size exclusion purification step (Superdex 200) in 100 mM NaCl, 10 mM NaF, 20 mM 4-(2-hydroxyethyl)piperazine-1-ethanesulfonic acid (HEPES) pH 7.5, 4 mM DM. To prepare proteoliposomes, 25 µg purified protein was mixed with 5 mg of *E. coli* polar lipid extract (Avanti Polar Lipids, Alabaster, AL) solubilized in 35 mM 3-[(3-cholamidopropyl)dimethylammonio]-1-propanesulfonate (CHAPS) and dialyzed against 0.3 M KF, 15 mM HEPES pH 7.0 at room temperature for 36 h with three buffer changes. Liposomes were stored in aliquots at −80°C until the day of use.

### Protein crosslinking

0.2 mg/mL purified protein was incubated with 0.125% glutaraldehyde (~500-fold molar excess) at room temperature. The reaction was quenched with 0.15 M Tris-HCl, pH 7.5 (10-fold molar excess) prior to analysis by SDS-PAGE.

### Fluoride efflux from proteoliposomes

Experiments were performed as described in detail (38). Briefly, after three freeze/thaw cycles, the multilamellar vesicles were extruded 21 times through a 400 nm membrane filter to form proteoliposomes. Liposomes were passed through a 1.5 mL Sephadex G20 resin column equilibrated with 0.3 M potassium isethionate, 1 mM KF, 15 mM HEPES pH 7.0, and diluted 20-fold in assay buffer in a stirred chamber. Extraliposomal fluoride was monitored with a fluoride electrode (Cole Palmer, USA) attached to a pH meter and digitized at a sampling frequency of 5 Hz. Transport was initiated by the addition of 1 µM of the potassium ionophore valinomycin to relieve the electrical potential and permit F^-^ and K^+^ to flow down their chemical gradients. At the end of the experiment, 30 mM octyl-β-D-glucopyranoside was added to release the remaining encapsulated fluoride. Chloride efflux experiments were performed similarly, except at pH 5.0 (a standard pH for bacterial chloride-transporting CLCs; buffered with 15 mM potassium citrate) using an Ag/AgCl electrode.

### Statistical analyses

Every experiment was performed as three independent biological replicates (*n* = 3). Two-way analysis of variance (ANOVA) using “species” and “fluoride” as the two factors, followed by Fisher’s LSD test was performed in GraphPad Prism 10 to calculate the standard error (SE) and statistical significance of the independent conditions.

## RESULTS

### Survey of fluoride export genes among the oral streptococci

We first examined the genetic context of fluoride export proteins found among streptococcus species (Fig. 1B and C). Primary colonizers of the oral cavity from the phylogenetically related mitis and sanguinis groups, which are usually associated with oral health, possess paired Fluc genes, which typically encode heterodimeric fluoride ion channels (39). By contrast, other oral streptococci, including pathogens *S. mutans* and *S. sobrinus* and other species from the mutans, anginosus, salivarius, and downei groups, instead possess CLC^F^-type F^−^/H^+^ antiporters (Table 1). Species in the mutans group feature two adjacent CLC^F^ open reading frames (1289 and 1290 in *S. mutans*) that encode proteins with ~60% sequence identity. The genetic context of the fluoride exporters is the same for both Fluc and CLC^F^ exporters and across streptococci, with the fluoride exporter gene(s) flanked by open reading frames for chorismate mutase on the 5′ end, and by ribosomal protein rplS on the 3′ end in most species.

**TABLE 1:** Fluoride exporters of the oral streptococci

Group	Example species	Fluoride exporter	Refseq protein ID
Mitis	*Streptococcus mitis* *Streptococcus oralis*	Fluc heterodimer Fluc heterodimer	WP_049500397.1,WP_000712114.1 WP_084881387.1, WP_084881388.1
Sanguinis	*Streptococcus sanguinis* *Streptococcus gordonii*	Fluc heterodimer Fluc heterodimer	WP_002933579.1,WP_002933586.1 WP_061603755.1,WP_061603756.1
Anginosus	*Streptococcus anginosus*	CLC^F^ (single)	WP_268730273.1 (lacks adjacent rplS)
Mutans	*Streptococcus mutans* UA159	CLC^F^ (paired)	WP_002263156.1, WP_002263157.1
Salivarius	*Streptococcus salivarius* *Streptococcus sobrinus*	CLC^F^ (single) CLC^F^ (single)	WP_014633220.1 WP_002962072.1
Downei	*Streptococcus downei*	CLC^F^ (single)	WP_002997240.1

### Fluoride is bactericidal for ∆CLC^F^ *S. mutans*

**Fig 2:**
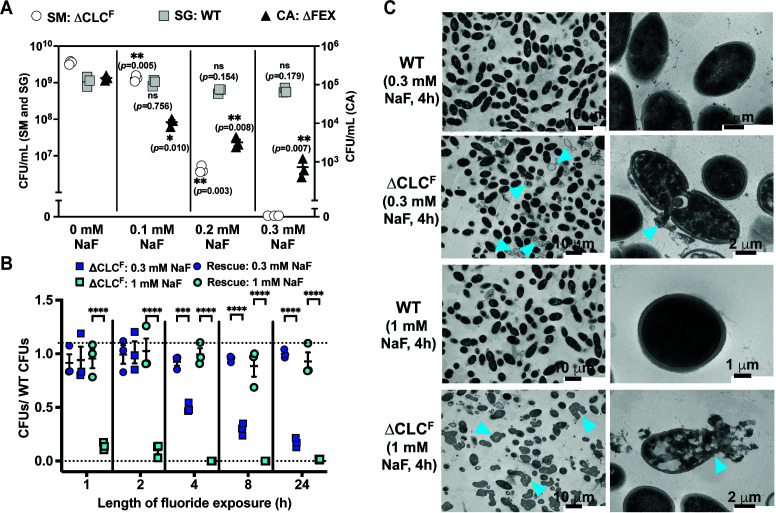
Effect of fluoride on the viability of ∆CLC^F^ *S. mutans* and ∆FEX *C. albicans*. (A) CFUs harvested from three-species surface biofilms (WT *S. gordonii*, ∆CLC^F^ *S. mutans*, ∆FEX *C. albicans*) after 24 h. Fluoride concentration (0, 0.1, 0.2, or 0.3 mM NaF) indicated. For biofilms grown in the presence of 0.1–0.3 mM NaF, significance is shown relative to the 0 NaF condition. Datapoints represent the mean and SEM of three independent experiments (*n* = 3). Significance was calculated using a two-way analysis of variance (ANOVA) followed by Fisher’s LSD test. (B) Recovered CFUs for ∆CLC^F^ *S. mutans* (squares) or ∆CLC^F^ *S. mutans* + pDL278 with CLC^F^ (circles) as a function of time, expressed as a fraction relative to WT *S. mutans*. Samples were treated with 0.3 NaF (dark blue) or 1 mM NaF (cyan). WT *S. mutans* CFUs are shown in Fig. S1. Datapoints represent the mean and SEM of three independent experiments (*n* = 3). Significance was calculated using a two-way analysis of variance (ANOVA) followed by Fisher’s LSD test. “****” represents *p* < 0.0001. (C) TEM images of ∆CLC^F^ or WT *S. mutans* cells after exposure to 0.3 or 1 mM NaF for 4 h. Blue arrows indicate examples of ghost cells due to leak of cytoplasmic contents (0.3 mM NaF, zoomed out view), dispersed cytosolic-nucleoid material due to complete cell wall digestion (1 mM NaF, zoomed out view), or ruptures in the cell wall (zoomed in views).

In agreement with the previous literature, fluoride exporter deletion strains for *S. mutans* (deletion of both the 1289 and 1290 open reading frames, referred to as ΔCLC^F^) and for *C. albicans* (homozygous FEX deletion, referred to as ΔFEX) exhibit sensitivity to fluoride. In our experiments, *C. albicans* and *S. mutans* both exhibit reduced growth by two log units in the presence of 0.1 mM NaF, and fail to grow at all in the presence of 0.3 mM NaF. The WT strains, as well as WT *S. gordonii*, are unaffected at these fluoride concentrations (Fig. 1D). Whereas these experiments, and those of previous studies (18, 19), monitored the growth of isolated strains on rich media, in the physiological context, microbes exist in complex biofilm communities. Biofilms can confer protection against environmental stressors but are also the site of extensive interspecies competition. To evaluate the fluoride sensitivity of the pathogenic strains in this context, we grew the ∆CLC^F^ *S. mutans* and ΔFEX *C. albicans* strains in three-species surface biofilms together with oral commensal *S. gordonii*, under conditions of increasing fluoride (Fig. 2A). After 24 h, biofilms were harvested and replated on fluoride-free recovery plates to determine the viable CFUs of each species after fluoride treatment. Similar to the previous dilution experiment, increasing doses of fluoride between 0.1 and 0.3 mM (5.7 ppm F^−^) significantly reduced ΔFEX *C. albicans* and ΔCLC^F^ *S. mutans* CFUs harvested from the mature biofilm, whereas the number of WT *S. gordonii* CFUs remained unaffected. Exposure to 0.2 mM (3.8 ppm F^−^) fluoride reduced ∆CLC^F^ *S. mutans* and ∆FEX *C. albicans* colony counts by 830- and 50-fold, respectively, compared to biofilms grown without fluoride treatment (Fig. 2A). For ∆FEX *C. albicans*, increasing fluoride to 0.3 mM (5.7 ppm) caused an additional 4.3-fold decrease in CFUs compared with those recovered from the biofilm exposed to 0.2 mM NaF (a 200-fold decrease compared to biofilms without fluoride). This agrees with the conclusion of a previous study that, although fluoride treatment interrupts *C. albicans* growth, a fraction of the population remains viable and can resume growth after the fluoride challenge is removed (18). In striking contrast, we observed no viable ∆CLC^F^ *S. mutans* colonies from biofilms treated with 0.3 mM fluoride for 24 h. These results suggested that ∆CLC^F^ *S. mutans* is at a severe competitive disadvantage and perhaps killed by relatively low concentrations of fluoride.

To explicitly test whether fluoride treatment is bactericidal for ∆CLC^F^ *S. mutans*, we exposed planktonic cultures of WT and ∆CLC^F^ *S. mutans* to 0.3 or 1 mM NaF for increasing lengths of time before plating on fluoride-free recovery plates. For cells treated with 0.3 mM NaF, the number of viable ∆CLC^F^ *S. mutans* colonies decreased as a function of time between 4 and 24 h. By contrast, when the gene encoding CLC^F^ was re-introduced on a plasmid, this strain remained viable after fluoride treatment for the entire timecourse Fig. 2B. This effect was even more striking when cultures were treated with 1 mM NaF. After just 1-h exposure, viable ∆CLC^F^ *S. mutans* CFUs were reduced 6.8-fold relative to WT (Fig. 2B). No survival of ∆CLC^F^ *S. mutans* was detected at timepoints ≥ 4 h of treatment. As for the 0.3 mM experiments, complementation with a plasmid-encoded CLC^F^ enabled the WT-like survival of the ∆CLC^F^ *S. mutans* strain. Indeed, for the WT and rescue strains, CFUs actually increased up to 24 h, indicating continued cell division (Fig. S1). Transmission electron microscopy (TEM) imaging showed widespread cell lysis and cell wall damage for ∆CLC^F^ *S. mutans* exposed to 0.3 mM NaF for 4 h, and lysis/total cell wall degradation of essentially all ∆CLC^F^ cells exposed to 1 mM NaF for 4 h (Fig. 2C). WT cells exhibited normal morphology after both treatments. Together, these experiments show that, if unable to export fluoride, *S. mutans* is killed and eliminated from biofilms by fluoride in the hundreds of micromolar range. The population of ∆FEX *C. albicans* is reduced by ~two orders of magnitude by fluoride treatment in this range, but not eliminated.

### Fluoride sensitivity in *S. mutans* synergistically reduces the *C. albicans* population in mixed-species biofilms

We next examined the effects of a single species’ fluoride sensitivity on the entire three-species community. For these experiments, we grew surface biofilms for 24 h in the presence of 0.2 mM NaF, and then measured the CFUs of each species, total biofilm mass, and media pH. We selected this concentration of fluoride because of the ∆CLC^F^ *S. mutans* and ∆FEX *C. albicans* knockout strains exhibit fluoride sensitivity, without being eliminated from the biofilm. As a negative control, we performed parallel biofilm experiments in the presence of 0.2 mM NaCl.

**Fig 3:**
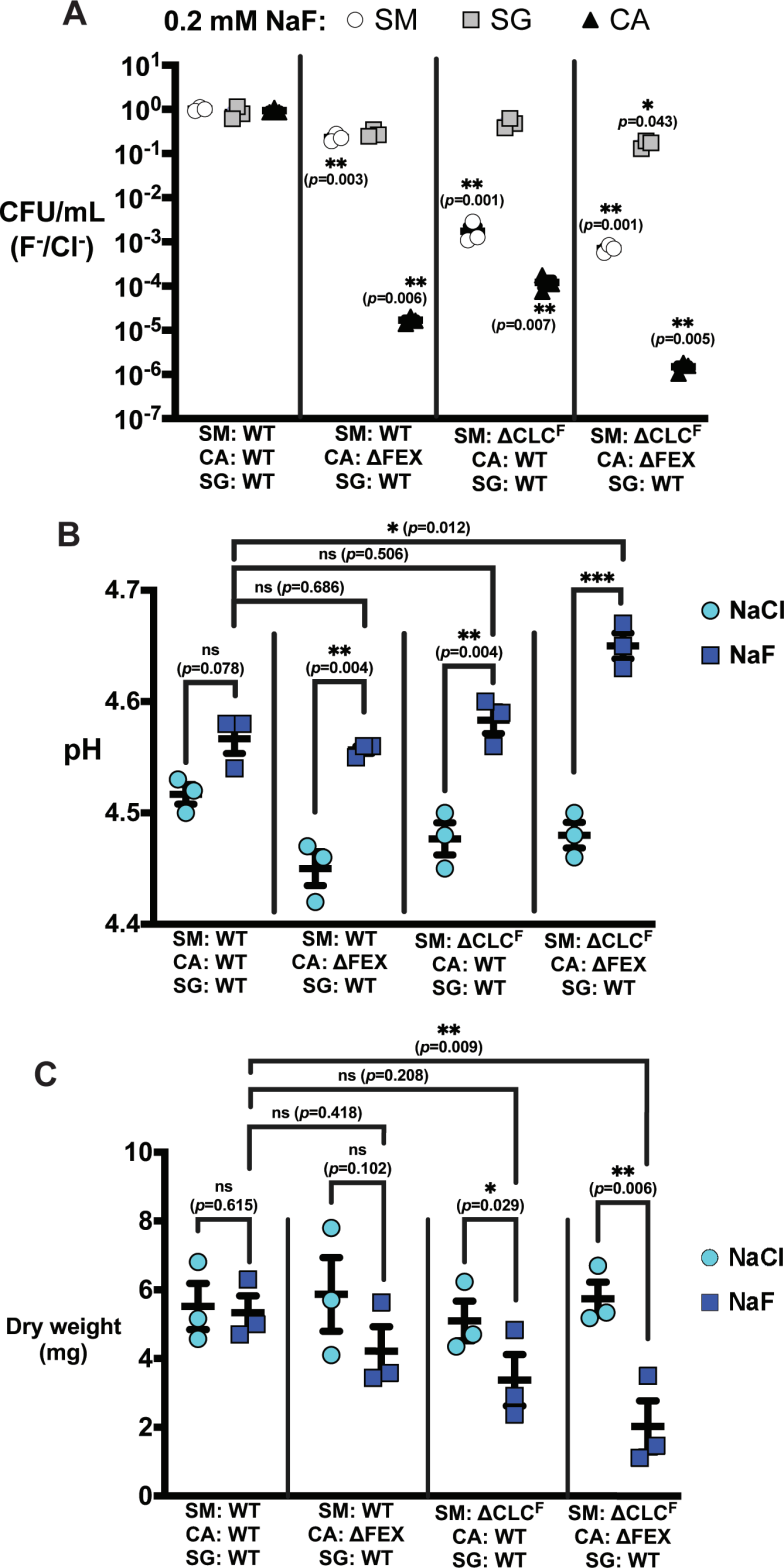
Properties of three-species biofilms grown with ∆CLC^F^ *S. mutans* or ∆FEX *C. albicans* in the presence of 0.2 mM NaF. (A) CFUs from fluoride-exposed biofilms expressed as a ratio relative to those obtained from control chloride-exposed biofilms. Unnormalized counts from the fluoride-treated and control biofilms are shown in Fig. S2. Significance (*p* < 0.05) is shown relative to the ratio of CFUs obtained from the all-WT biofilms. (B) Media pH after 24-h biofilm growth. C. Biofilm mass (dry weight) after 24-h growth. For all panels, datapoints represent the mean and SEM of three independent experiments (*n* = 3). Significance was calculated using a two-way analysis of variance (ANOVA) followed by Fisher’s LSD test. Statistical significance is represented as *p < 0.05, **p < 0.01, and ***p < 0.001.

Unsurprisingly, in biofilms with a single fluoride-sensitive species, that species was significantly reduced in fluoride-containing media relative to chloride. However, we also observed a synergistic reduction in CFUs of the second cariogenic species, especially for biofilms grown with ∆CLC^F^ *S. mutans* (Fig. 3A; Fig. S2; Table S2). In these three-species biofilms, with WT *C. albicans*, WT *S. gordonii*, and ∆CLC^F^ *S. mutans*, the *S. mutans* population was reduced by a factor of 500, and the WT *C. albicans* population by a factor of 7,000 compared to the chloride control. Strikingly, this reduction in the *C. albicans* population upon knockout of the *S. mutans* fluoride exporter is almost as great as the reduction in *C. albicans* population due to knockout of its fluoride exporter, 5 × 10^4^ fold. This is consistent with the role of *S. mutans* in extracellular aggregation and biofilm stabilization, which promotes the growth of symbionts like *C. albicans* (4, 8). For the three-species biofilms composed of ∆FEX *C. albicans,* WT *S. mutans*, and WT *S. gordonii*, in addition to the reduction of ∆FEX *C. albicans* described above, WT *S. mutans* population decreased by a small, but significant factor of 4.4 compared to the chloride control. In both cases, no significant change in the *S. gordonii* population was observed. Only when biofilms were grown with both ∆FEX *C. albicans* and ∆CLC^F^ *S. mutans* was there a significant decrease in the *S. gordonii* population compared to the chloride control. As for the *C. albicans* result described above, we attribute the decreased *S. gordonii* population to an overall reduction in microbial growth due to diminished extracellular matrix production by *S. mutans*.

For all biofilms with ∆FEX *C. albicans,* ∆CLC^F^ *S. mutans*, or both, fluoride treatment significantly increased the pH of the biofilm media relative to the NaCl control. However, among the fluoride-treated biofilms, only the samples with both ∆CLC^F^ *S. mutans* and ∆FEX *C. albicans* showed significantly higher media pH compared to biofilms with the WT counterparts (Fig. 3B). Both biofilms with ∆CLC^F^ *S. mutans* exhibited significantly lower dry-weight biomass compared to all-WT or chloride-treated controls, in accord with *S. mutans*’ role as a keystone pathogen whose fitness impacts biofilm stability and assembly (Fig. 3C). Together, these experiments demonstrate that fluoride sensitivity in pathogens, especially *S. mutans*, impact biofilm species composition, mass, and acidogenicity.

### The importance of fluoride export to fitness is recapitulated in biofilms exposed to feast-and-famine cycles

**Fig 4:**
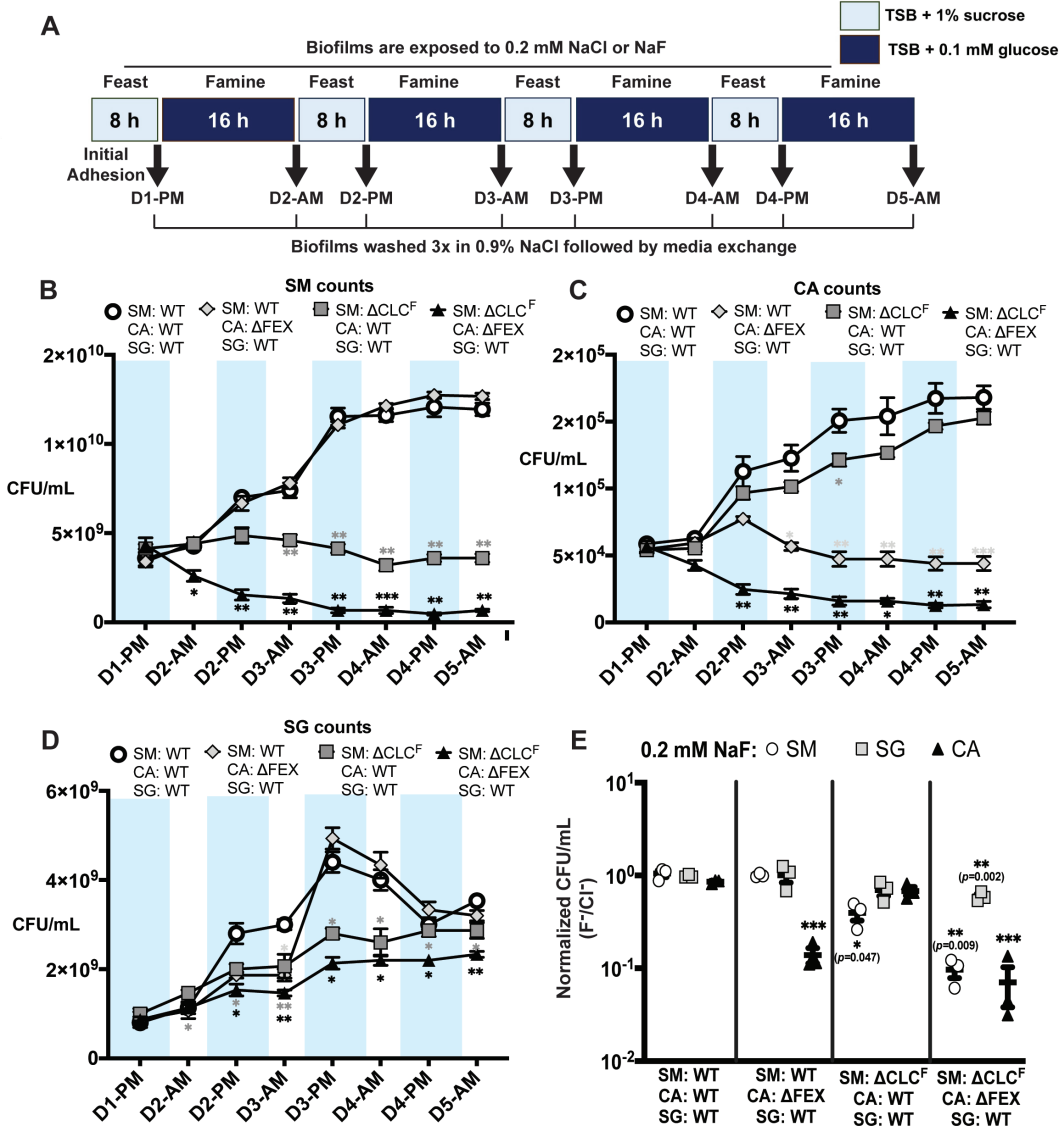
Species composition of biofilms grown on hydroxyapatite discs with feast/famine cycles. (A) Schematic of media changes for the feast-and-famine dental biofilm model. (B) Final counts of CFUs for each microbial species at the end of the 5-day experiment. CFUs for biofilms grown in the presence of 0.2 mM NaF are normalized relative to biofilms grown in the presence of 0.2 mM NaCl. Unnormalized counts from the fluoride-treated and control biofilms are shown in Fig. S3. (C) *S. mutans* CFUs recovered from biofilms of the indicated composition after each treatment in the feast and famine cycle. Statistical significance is shown relative to all-WT fluoride-treated biofilms (open circles). (D). *C. albicans* CFUs recovered from biofilms of the indicated composition after each treatment in the feast and famine cycle. Statistical significance is shown relative to all-WT biofilms and fluoride-treated biofilms (open circles). (E) *S. gordonii* CFUs recovered from biofilms of the indicated composition after each treatment in the feast-and-famine cycle. Statistical significance is shown relative to all-WT biofilms and fluoride-treated biofilms (open circles). For all panels, datapoints represent the mean and SEM of three independent experiments (*n* = 3). Significance was calculated using a two-way analysis of variance (ANOVA) followed by Fisher’s LSD test. Statistical significance is shown relative to parallel experiments in the presence of 0.2 mm NaCl and is represented as *p < 0.05, **p < 0.01, and ***p < 0.001.

The experiments described above suggest that biofilms composed of fluoride-sensitive strains will be less pathogenic than WT biofilms. To evaluate this in a more faithful model of dental caries, we next grew biofilms on hydroxyapatite, the main chemical component of tooth enamel. For these experiments, we exposed the biofilms to feast-and-famine periods that mimic the cycles of daytime sucrose consumption and overnight fasting experienced by oral microbiota (Fig. 4A)(40). We exposed the biofilms to the same sub-lethal concentration of 0.2 mM NaF (or 0.2 mM NaCl as a control) after the biofilm was established, beginning on day 2, and examined the biofilm species composition after each feast and famine period for 5 days (Fig. 4B through D). For all-WT biofilms, the counts of *S. mutans* and *C. albicans* both increased over the course of the experiment, reaching a steady state by day 5. By contrast, *S. gordonii* counts peaked on day 3, before being outcompeted by the other two species. When biofilms were prepared with ΔFEX *C. albicans*, the overall trend of *S. mutans* and *S. gordonii* growth was preserved, along with the expected reduction in ΔFEX *C. albicans* over the course of the experiment. However, for biofilms prepared with ΔCLC^F^ *S. mutans*, populations of other species were impacted as well. Although the *C. albicans* population increased over the course of the experiment, there was a small reduction in total counts relative to its growth in chloride-exposed biofilms (*P*-values between 0.031 and 0.221 at each timepoint beyond day 2). The *S. gordonii* population also increased over the course of the experiment, reaching a similar population at day 5 as in the all-WT or ΔFEX *C. albicans* experiments, but without the population peak and collapse observed in those two samples. A similar trend in *S. gordonii* growth was also present in the biofilm sample with both ΔFEX *C. albicans* and ΔCLC^F^ *S. mutans*. As in the surface biofilms, synergy between *S. mutans* and *C. albicans* growth was observed, as counts for both species were significantly reduced in biofilms with both knockout strains, compared with biofilms with either individual knockout strain (Fig. 4B through E; Fig. S3).

### Fluoride sensitivity in *S. mutans* or *C. albicans* reduces the pathogenic potential of the biofilm

**Fig 5:**
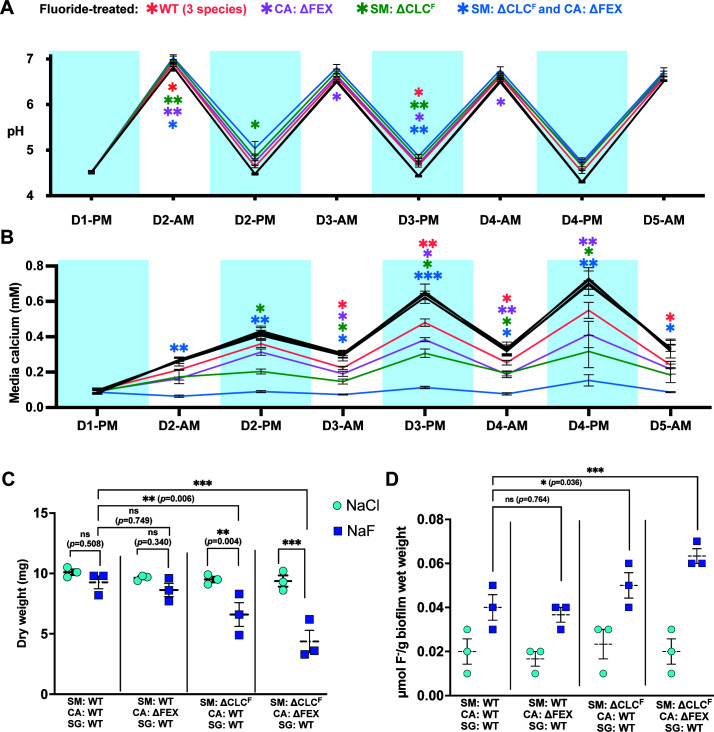
Properties of biofilms grown on hydroxyapatite discs with feast/famine cycles. (A) Media pH was measured after each cycle of feast and famine. (B) Calcium concentration in media was measured after each cycle of feast and famine. For panels A and B, for each biofilm composition, control experiments were performed in the presence of NaCl (black points/lines in panels A and B). These show close correspondence with overlapping points. For the fluoride-treated conditions, statistical significance is shown relative to the chloride-treated biofilms and is represented as *p < 0.05, **p < 0.01, and ***p < 0.001. (C) Final biofilm mass at the end of the 5-day experiment. (D). Fluoride content of biofilm at the end of the 5-day experiment. For all panels, datapoints represent the mean and SEM of three independent experiments (*n* = 3). Significance was calculated using a two-way analysis of variance (ANOVA) followed by Fisher’s LSD test.

We also examined several indicators of disease progression over the course of the multiday experiment, including media pH and calcium release from the hydroxyapatite (Fig. 5A and B), and, at the end of the experiment, biofilm mass and biofilm fluoride content (Fig. 5C and D). These measurements show pH oscillations characteristic of the feast and famine periods. Drastic reductions in pH occur during every feast period due to sucrose fermentation, and much smaller reductions in pH are seen during the overnight famine period because the limiting glucose concentration minimizes fermentation. The pH at the end of the feast period was consistently higher throughout the experiment for fluoride-treated biofilms in which either of the pathogenic microorganisms was fluoride export deficient (Fig. 5A; Table 2; Table S3). The lowest acid production was observed in the fluoride-treated biofilms grown with mutant strains of both *S. mutans* and *C. albicans*. Fluoride-treated biofilms prepared with both fluoride-sensitive strains exhibited a significant reduction in acid production (increased pH) even after the famine periods, which already entails less acid production due to reduced sugar availability.

**TABLE 2:** Variations in pH in the feast-and-famine model^*a*^

	WT		Ca (∆FEX)		Sm (∆CLC^F^)		Sm (∆CLC^F^)- Ca (∆FEX)	
	**NaCl**	**NaF**	**NaCl**	**NaF**	**NaCl**	**NaF**	**NaCl**	**NaF**
D1-PM	4.51 ± 0.02	4.51 ± 0.02	4.50 ± 0.03	4.51 ± 0.02	4.52 ± 0.03	4.50 ± 0.01	4.52 ± 0.03	4.51 ± 0.02
D2-AM	6.83 ± 0.06	6.91 ± 0.08*	6.83 ± 0.07	6.93 ± 0.06**	6.82 ± 0.08	7.00 ± 0.06**	6.83 ± 0.09	7.03 ± 0.06*
D2-PM	4.47 ± 0.02	4.64 ± 0.03	4.47 ± 0.00	4.75 ± 0.06	4.47 ± 0.01	4.84 ± 0.07*	4.48 ± 0.02	5.04 ± 0.15
D3-AM	6.52 ± 0.03	6.59 ± 0.00	6.49 ± 0.01	6.64 ± 0.02*	6.52 ± 0.03	6.73 ± 0.05	6.52 ± 0.02	6.81 ± 0.07
D3-PM	4.44 ± 0.01	4.66 ± 0.03*	4.43 ± 0.01	4.71 ± 0.04*	4.43 ± 0.01	4.79 ± 0.03**	4.44 ± 0.01	4.87 ± 0.04**
D4-AM	6.55 ± 0.03	6.61 ± 0.02	6.49 ± 0.02	6.64 ± 0.04*	6.53 ± 0.02	6.68 ± 0.04	6.53 ± 0.02	6.76 ± 0.07
D4-PM	4.31 ± 0.02	4.52 ± 0.03	4.29 ± 0.00	4.70 ± 0.10	4.28 ± 0.00	4.65 ± 0.08	4.30 ± 0.01	4.73 ± 0.10
D5-AM	6.53 ± 0.02	6.58 ± 0.05	6.51 ± 0.01	6.61 ± 0.07	6.52 ± 0.01	6.67 ± 0.07	6.51 ± 0.01	6.72 ± 0.09

^*a*^ For all data, datapoints represent the mean and SEM of three independent biological replicates (*n* = 3). Significance was calculated using a two-way analysis of variance (ANOVA) followed by Fisher’s LSD test implemented in GraphPad Prism 10. Statistical significance is represented as **p < 0.05, **p < 0.01, and ***p < 0.001.

Similar to pH, we observed oscillations in the media calcium content corresponding to the periods of feast and famine since acid produced during sucrose fermentation accelerates the dissolution of the hydroxyapatite discs (41). Fluoride helps prevent demineralization *via* a physicochemical effect, so even all-WT biofilms showed lower calcium release into the media compared to the chloride-treated biofilms. However, the biofilms prepared with fluoride-sensitive strains exhibited even lower levels of hydroxyapatite dissolution. Temporal release of calcium was the lowest in the fluoride-treated biofilms grown from the combination of the mutant strains of both pathogens, with statistically significant differences compared to the chloride-treated biofilms emerging at all timepoints after fluoride addition, which occurred after the famine treatment on the second day (D2-AM) (Fig. 5B). The biofilms with ∆CLC^F^ *S. mutans* or ΔFEX *C. albicans* also exhibited significantly lower hydroxyapatite dissolution at most timepoints. The observation of decreased demineralization is intertwined with the reductions in pathogen CFUs and reduced acid production already discussed. These results show that, in clinically relevant conditions, fluoride sensitivity of the biofilm’s constituent species has downstream effects on several markers of dental caries.

As in the surface biofilms, the biomass of the mature biofilm was significantly reduced for fluoride-treated biofilms prepared with ∆CLC^F^ *S. mutans*, compared to the respective chloride-treated biofilms (Fig. 5C). Interestingly, these biofilms also accumulate the highest levels of fluoride (Fig. 5D). We propose that this is due to fluoride accumulation in the cytoplasm of the species that lack a fluoride exporter, as has been shown to occur in other fluoride export-deficient bacteria (17, 20). When the fluoride concentration is increased to the level that causes lysis and elimination of *S. mutans*, 0.3 mM, fluoride accumulation in the biofilm is likewise eliminated (Fig. S4).

### Only one open reading frame contributes to the functional CLC^F^ exporter in *S. mutans*

The experiments thus far suggest that the *S. mutans* CLC^F^ is essential to the stability of the cariogenic dental biofilm under conditions of fluoride exposure. Thus, we sought to determine whether ORF 1289, ORF 1290, or both genes encode the functional, fluoride-exporting unit. Across kingdoms of life, CLC proteins form functional homodimers (42), but we also considered the possibility that the *S. mutans* CLC^F^ might be a heterodimer of both gene products. While the contribution of both genes to fluoride resistance has been examined for *S. mutans* UA159 previously, different studies have come to different conclusions about whether 1289 only (19), or both 1289 and 1290 (30), contribute to fluoride efflux. Sequence alignments indicate that both 1289 and 1290 possess the characteristic fluoride-binding motifs observed in structures of the CLC^F^ from *Enterococcus casseliflavus* (26) (Fig. S5), which are quite distinct from the selectivity filter sequences of CLC chloride transporters (22, 43).

**Fig 6:**
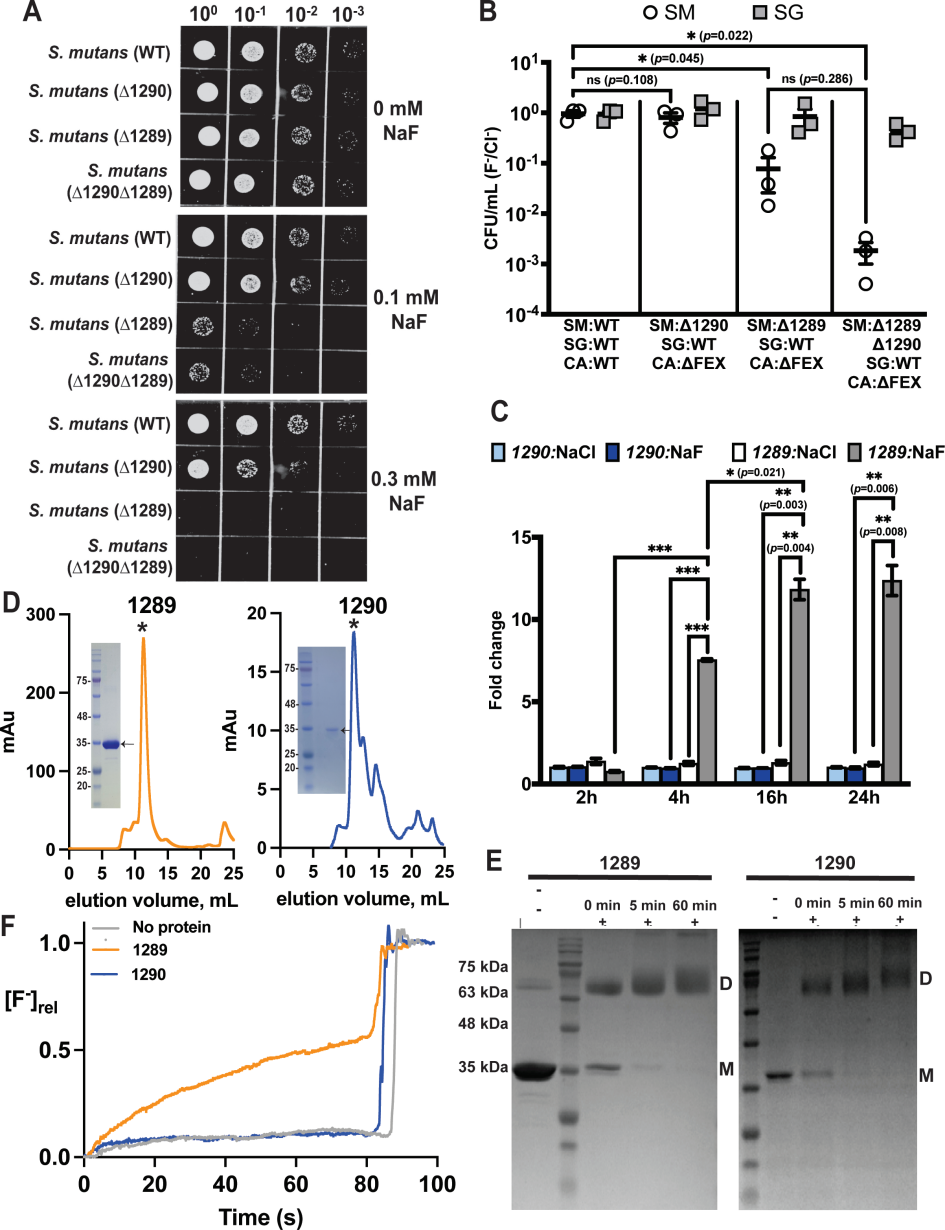
Contributions of ORF 1289 and ORF 1290 to fluoride resistance and transport in *S. mutans*. (A) 10-fold serial dilutions of ∆1289 *S*. *mutans*, ∆1290 *S*. *mutans,* and the ∆CLC^F^ strain (∆1290∆1289) used in prior experiments. Plates contain NaF as indicated. (B) CFUs of *S. mutans* and *S. gordonii* harvested from three-species (*S. mutans, S. gordonii,* and *C. albicans*) biofilms after 24-h growth in the presence of 0.2 mM NaF, normalized relative to CFUs harvested from biofilms grown in the presence of 0.2 mM NaCl. Significance is expressed relative to the corresponding biofilm grown with 0.2 mM NaCl and was calculated using two-way analysis of variance (ANOVA) followed by Fisher’s LSD test. (C) Gene expression profile of *S. mutans* ORFs 1290 and 1289 in three-species biofilms (WT *S. mutans*) grown for variable lengths of time. Gene expression is shown as fold-change relative to housekeeping gene *gyrA*. Datapoints represent the mean and SEM of three independent biological replicates (*n* = 3). Significance was calculated using a two-way analysis of variance (ANOVA) followed by Fisher’s LSD test. Independent control experiments verified gene amplification by both sets of primers (Fig. S7). (D) Size exclusion chromatograms (SEC) of purified proteins encoded by ORF 1289 (left) and ORF 1290 (right). The asterisks indicate the peak that was collected and reconstituted for functional analysis. Insets: SDS-PAGE gel of major SEC peak stained with Coomassie. Note that membrane proteins often run at a lower molecular weight than expected due to incomplete denaturation in SDS detergent. (E) Crosslinking analysis of proteins from panel D. Samples were exposed to 0.125% glutaraldehyde for the indicated time. Bands consistent with a monomer and dimer are indicated by the labels “M” and “D,” respectively. (F) Fluoride efflux from liposomes reconstituted with 1289 (orange), 1290 (blue), or no protein (gray). Fluoride transport is initiated by valinomycin addition at 5 s. Traces are normalized relative to total encapsulated F^−^, which is determined after fluoride addition (sharp increase in F^-^ at ~80 s). Traces are representative of data from three independent proteoliposome preparations. The rate of transport was 830 ± 20 F^−^/s for ORF 1289 and 17 ± 5 F^-^/s for ORF 1290 (mean and SEM from three liposome preparations).

We first performed bacterial dilution assays for *S. mutans* strains with ORF 1289 and ORF 1290 knocked out individually (referred to as ∆1289 and ∆1290, respectively). ∆1289 *S*. *mutans* exhibited comparable fluoride sensitivity to the ∆CLC^F^ *S. mutans* strain used for earlier experiments, in which both open reading frames are deleted. By contrast, growth of ∆1290 *S*. *mutans* was not affected at 0.1 mM NaF, similar to the WT strain, and showed only a marginal sensitivity to 0.3 mM NaF (Fig. 6A). These data suggest that ORF 1289 contributes more toward the fluoride resistance phenotype.

We next tested the hypothesis that ORF 1290 is expressed at a different timepoint or under different nutrient conditions inherent to biofilm growth. We examined ∆1289, ∆1290, and the double knockout in three-species surface biofilms with *S. gordonii* and *C. albicans*. In agreement with the dilution assays*,* ∆1289 exhibited significantly reduced survival in fluoride compared to chloride, comparable to the ∆CLC^F^ strain with deletions of both ORFs 1290 and 1289. For biofilms grown with ∆1290, there was a slight, but not statistically significant, decrease in *S. mutans* CFUs harvested from the biofilm (*p* = 0.108; Fig. 6B; Fig. S6). Similarly, the ∆1289 ∆1290 double knockout was not significantly less fit relative to the ∆1289 single knockout in fluoride (*p* = 0.29; Fig. 6B).

Quantitative gene expression profiling of samples collected from biofilms formed with WT *S. mutans* corroborated these phenotypic observations. ORF 1289 showed an increase in transcripts over time when the biofilm was treated with 0.2 mM NaF, with a 17-fold increase between 1 and 24 h of fluoride exposure. By contrast, expression of ORF 1290 did not change over 24 h of fluoride treatment and remained at a similar level as ORFs 1290 and 1289 under chloride treatment (Fig. 6C).

To assess transport function, we purified the proteins encoded by the individual ORFs and tested their ability to export fluoride upon functional reconstitution in liposomes. Proteins encoded by both ORFs 1289 and 1290 could be heterologously expressed in *E. coli* under a tetracycline promoter and purified. Both proteins showed monodisperse peaks by size exclusion chromatography, an indication of protein stability, although the yield of 1290 was lower (Fig. 6D). Crosslinking experiments showed that both 1289 and 1290 purify as homodimers, similar to other CLC proteins, providing another indication that both proteins are properly folded and able to assemble (Fig. 6E). We reconstituted purified 1289 and 1290 in liposomes and monitored fluoride transport by each protein individually using a fluoride electrode-based transport assay (43). Western blot analysis showed that both 1289 and 1290 were incorporated into proteoliposomes at similar levels (Fig. S8). 1289 catalyzed robust fluoride efflux from liposomes, with a unitary transport rate of 830 ± 20 ions/second, in line with rates measured for other CLC^F^ transporters (43). By contrast, fluoride efflux from proteoliposomes containing 1290 was indistinguishable from uncatalyzed fluoride leak exhibited by protein-free liposomes (Fig. 6F). We further tested chloride efflux by 1290, but did not observe any transport activity for this halide either (Fig. S9). From these lines of evidence, resistance assays, quantitative transcript profiling, and functional reconstitution, we conclude that the functional *S. mutans* CLC^F^ is a homodimer encoded by ORF 1289.

## DISCUSSION

In this work, we sought to understand how fluoride export impacts the fitness of pathogens in dental biofilms, a question with particular relevance to the oral microbiota due to the widespread use of fluoride in oral healthcare products. Moreover, the observation that dental pathogens and oral eubacteria export fluoride using proteins from entirely different molecular families provides an opportunity to specifically target fluoride export by oral pathogens. We, therefore, examined the effects of fluoride export at the individual species level, in model three-species surface biofilms, and in biofilms grown on the dental mineral hydroxyapatite under conditions that mimic physiologically realistic cycles of feast and famine.

As expected from other studies of fluoride exporter deletion strains, both *S. mutans* and *C. albicans* are rendered sensitive to hundreds of micromolar F^−^ upon deletion of their respective fluoride exporters. Although biofilms are protective against many antimicrobial stressors, fluoride is inhibitory to a similar extent in a biofilm context. Particularly striking was the elimination of ∆CLC^F^ *S. mutans* from biofilms at a low fluoride concentration of 0.3 mM (5.7 ppm F^-^), a concentration almost 200-fold lower than the fluoride levels in toothpaste and just 8-fold higher than that in fluoridated municipal water supplies (44, 45). Whereas fluoride acts as a bacteriostatic agent for many microbes (18, 20, 46), ∆CLC^F^ *S. mutans* unexpectedly underwent massive lysis under fluoride stress. *S. mutans* is known to induce autolysins in response to stressors such as heat or protein synthesis inhibitors (47). Indeed, partial autolysis of WT *S. mutans* exposed to 5 mM NaF was reported decades ago (48). Typically autolysis is adaptive, eliminating a subpopulation of cells to provide nutrients or genetic material to sister cells. The mechanism of fluoride-induced lysis, and why lysis is not limited to a sub-population, remains to be explored.

Knockout of the *S. mutans* fluoride exporter influences the properties of the entire biofilm under fluoride stress. This finding is not unexpected since *S. mutans* actively contributes to dysbiosis by secreting enzymes that can polymerize sucrose into extracellular polysaccharides, enhancing biofilm mass and providing a substrate for other species growth (49); and further by producing acid that eliminates species that are not tolerant to low pH (50). Biofilms grown with ∆CLC^F^ *S. mutans* exhibited a lower *C. albicans* population, a greater proportion of *S. gordonii*, higher pH, and less demineralization. The total biofilm biomass was also decreased, as fluoride sensitivity in *S. mutans* did not cause overgrowth of *S. gordonii*, which infrequently causes infective endocarditis or other invasive infections (51). While biofilms prepared with ∆FEX *C. albicans* also exhibited lower pathogenicity, the effect was not as large as that of ∆CLC^F^ *S. mutans*.

An unexpected finding was the retention of fluoride in biofilms with ∆CLC^F^ *S. mutans*. This effect mirrors the intracellular fluoride accumulation that has been measured in other microbes that lack fluoride exporters (17, 20, 52). In these cells, intracellular fluoride accumulation quantitatively follows the pH gradient (20). For example, for cells in a pH 5 environment, with a cytoplasmic pH maintained at 7, fluoride will accumulate intracellularly to a concentration 100-fold greater than that of the external fluoride. For ∆CLC^F^ *S. mutans*, we expect that fluoride accumulation only occurs in living cells; at higher concentrations, we observe cell lysis, which would release fluoride from the biofilm where it can be washed away. This implies that at moderate fluoride concentrations, the bacteria themselves could act as fluoride reservoirs, slowly releasing fluoride into the biofilm over several hours as the population lyses, and exposing the tooth enamel to protective fluoride for some time after fluoride treatment.

Because of its primary importance to *S. mutans* fitness in fluoride-treated biofilms, we sought to determine the functional unit of the *S. mutans* CLC^F^. The literature features contrasting results, with one study concluding that both ORFs 1289 and 1290 contribute to fluoride resistance (30), and another study concluding that 1289 only was responsible for the fluoride resistance phenotype(19) (all studies investigated *S. mutans* UA159). Although the reason for the discrepancy in the previous studies is not clear, we found that 1290 contributed little to resistance, either in culture or in the biofilm context. Moreover, we did not observe fluoride-induced changes in the transcription of ORF 1290. However, we note that in a fluoride-resistant derivative of *S. mutans* UA159, mutations in the promoter region 5′ to 1290 contribute to constitutive expression of ORF 1290 and other genes in the operon including ORF 1289 (53). Thus, we cannot definitively rule out the possibility that in other closely related bacteria from the mutans subgroup, both genes are expressed. However, our functional studies suggest that even if 1290 is expressed, the resulting protein does not contribute to fluoride efflux. We were able to heterologously express and purify a well-folded protein encoded by 1290 under an artificial promoter, but it did not transport fluoride from reconstituted proteoliposomes. Sequence alignments show that most of the fluoride-binding residues identified in the structure of the *Enterococcus casseliflavus* CLC^F^ (26) are conserved in 1290, except T320 (*E. casseliflavus* numbering) (Fig. S5). In 1290, there is methionine at this position. In the *E. casseliflavus* protein, the T320A mutation inhibited transport (26), so we hypothesize that the mutation of this residue in *S. mutans* 1290 is responsible for its inactivity. Although ORF 1290 presumably originated from a duplication of a functional CLC^F^ gene, it appears that the redundant gene has degraded into a pseudogene that is not expressed or functional. 1289, in contrast, is induced by fluoride and encodes a homodimeric transporter that catalyzes efficient fluoride export from lipid vesicles. Based on the sum of this evidence, we conclude that in *S. mutans* UA159, ORF 1289 is necessary and sufficient for fluoride transport and resistance.

**Fig 7:**
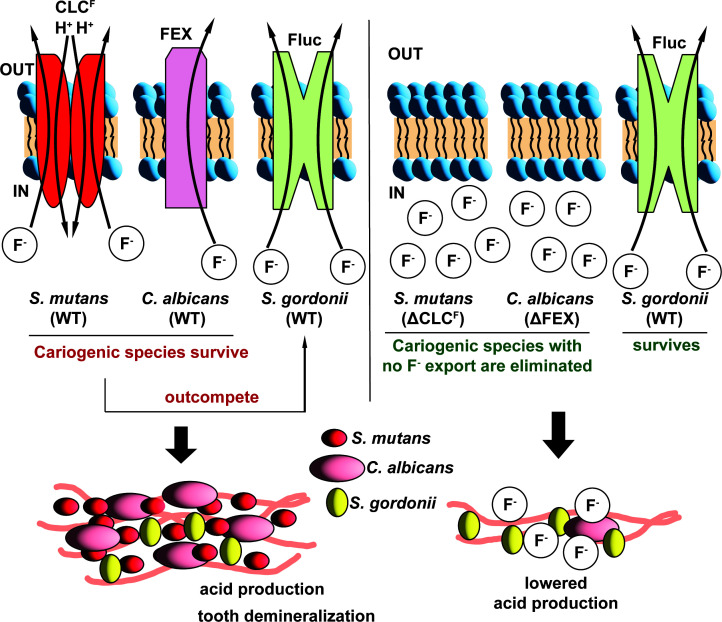
Schematic of species composition in dental biofilms. Left: all WT species. Right: species with deletion of fluoride export gene.

In summary, we report substantial changes in dental biofilm communities based on a single factor, fluoride export (Fig. 7). Our results imply that fluoride efflux inhibitors that specifically target the CLC^F^ of *S. mutans* or the FEX channel of *C. albicans* could serve to reduce or eliminate cariogenic species while preserving commensal microbes in the dental biofilm, which mainly export fluoride *via* the action of the molecularly distinct Fluc channels. Humans also lack known fluoride exporters, including CLC^F^ or FEX proteins, further recommending fluoride efflux as a potential antimicrobial target. The *S. mutans* CLC^F^ is a particularly attractive target for the development of such an inhibitor because of its sensitivity to lysis under fluoride stress, its keystone role in biofilm assembly and pathogenesis, and its straightforward architecture as a homodimer encoded by a single open reading frame.

## SUPPLEMENTAL MATERIAL

Supplemental material mbio.00184-24-s0001.pdf Figures S1-S9 and Tables S1-S3.

ASM does not own the copyrights to Supplemental Material that may be linked to, or accessed through, an article. The authors have granted ASM a non-exclusive, world-wide license to publish the Supplemental Material files. Please contact the corresponding author directly for reuse.
