# Supplementary material for: Fluoride export is required for the competitive fitness of pathogenic microorganisms in dental biofilm models
Source: mBio. 2024 Apr 16;15(5):e00184-24. doi: 10.1128/mbio.00184-24 (PMC11077948; doi:10.1128/mbio.00184-24)
Supplement: Supplemental material — Figures S1-S9 and Tables S1-S3. [file mbio.00184-24-s0001.pdf]

Supplementary Data

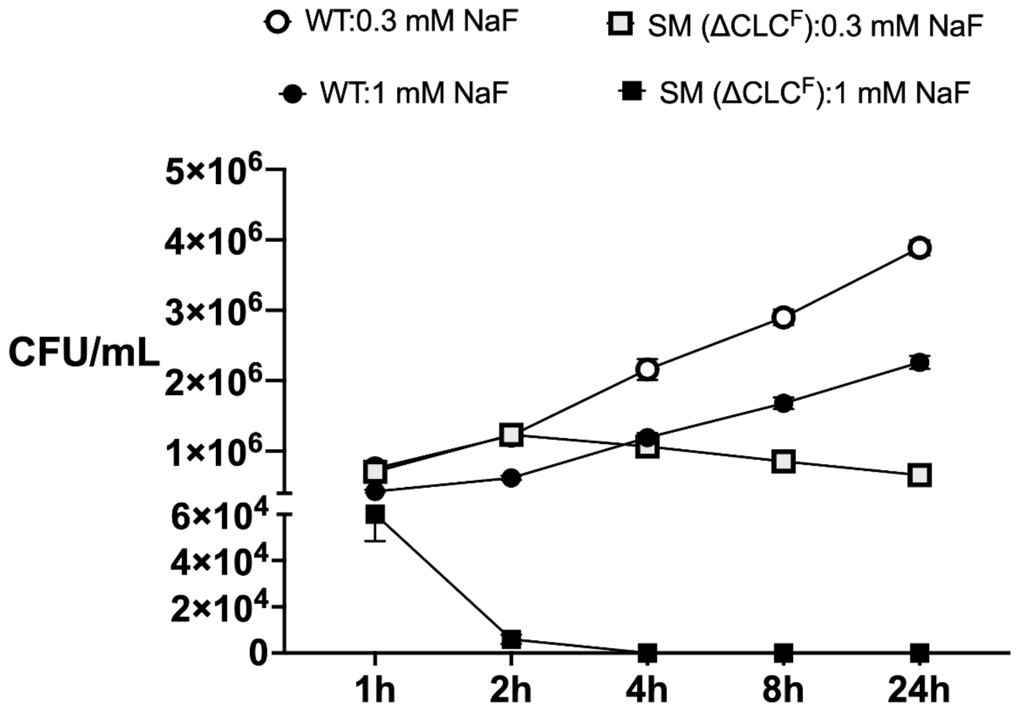

Supplementary Fig. 1. Recovered CFUs of ΔCLC^F and WT *S. mutans* as a function of time for samples treated with 0.3 or 1 mM NaF.

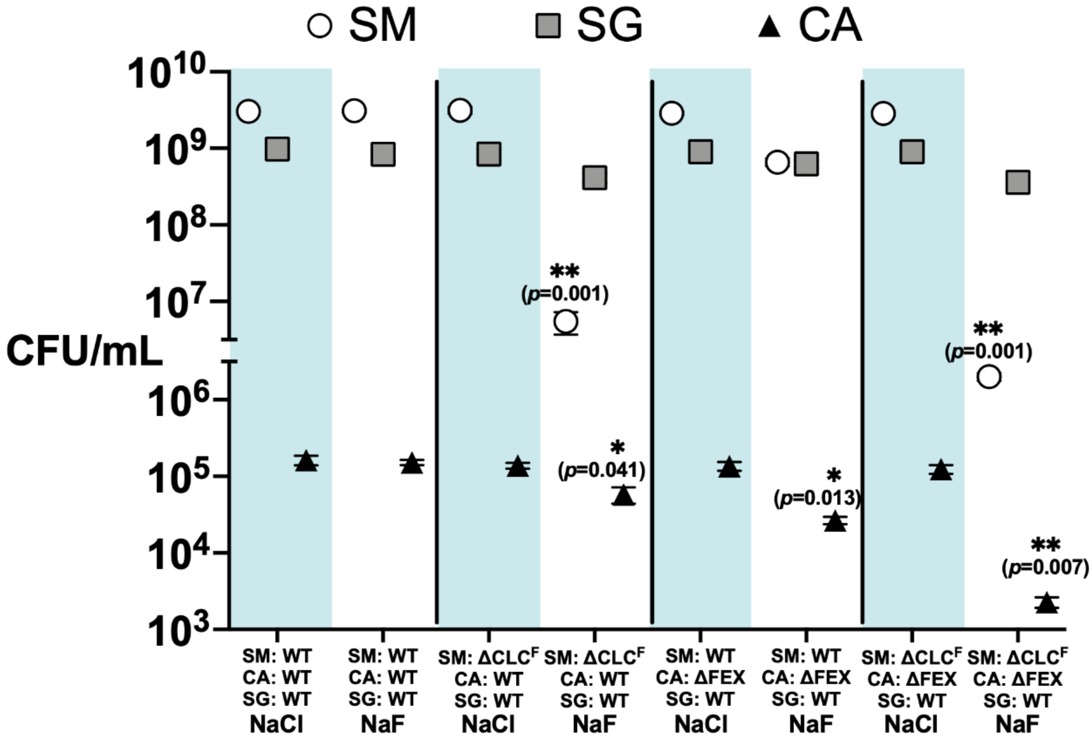

Supplementary Fig. 2. CFUs of *S. mutans*, *S. gordonii* and *C. albicans* recovered from three-species biofilms grown with ΔCLC^F *S. mutans* or ΔFEX *C. albicans* or respective WT strains in the presence of 0.2 mM NaCl or NaF, and harvested after 24 h. The datapoints represent mean and SEM of three independent experiments (n=3). Significance was calculated using two-way analysis of variance (ANOVA) followed by Fisher's LSD test. Statistically significant differences (p< 0.05) relative to the all-WT biofilms are indicated and represented as '*' (p< 0.05), '**' (p< 0.01) and '***' (p< 0.001).

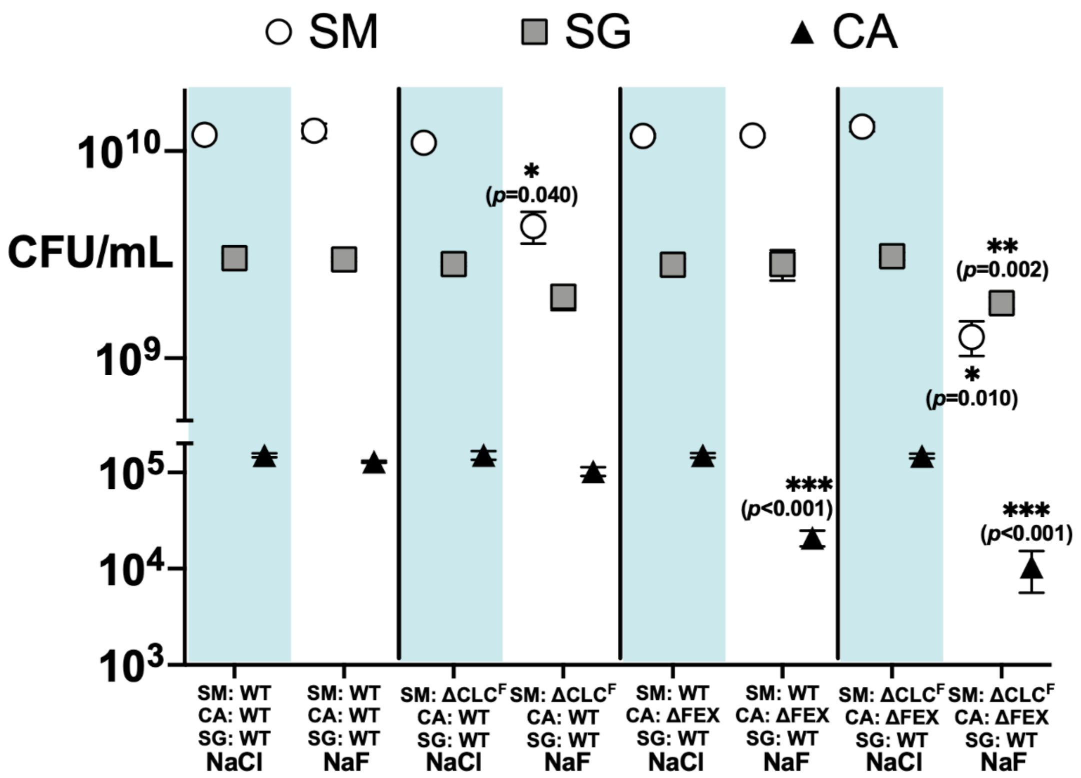

Supplementary Fig. 3. Final counts of CFUs for *S. mutans*, *S. gordonii* and *C. albicans* recovered from three-species biofilms grown with ΔCLC^F *S. mutans* or ΔFEX *C. albicans* or respective WT strains in the presence of 0.2 mM NaCl or NaF and at the end of the 5-day feast-and-famine experiment. The datapoints represent mean and SEM of three independent experiments (*n*=3). Significance was calculated using two-way analysis of variance (ANOVA) followed by Fisher's LSD test. Statistical significance is represented as '*' ($p<$ 0.05), '**' ($p<$ 0.01) and '***' ($p<$ 0.001).

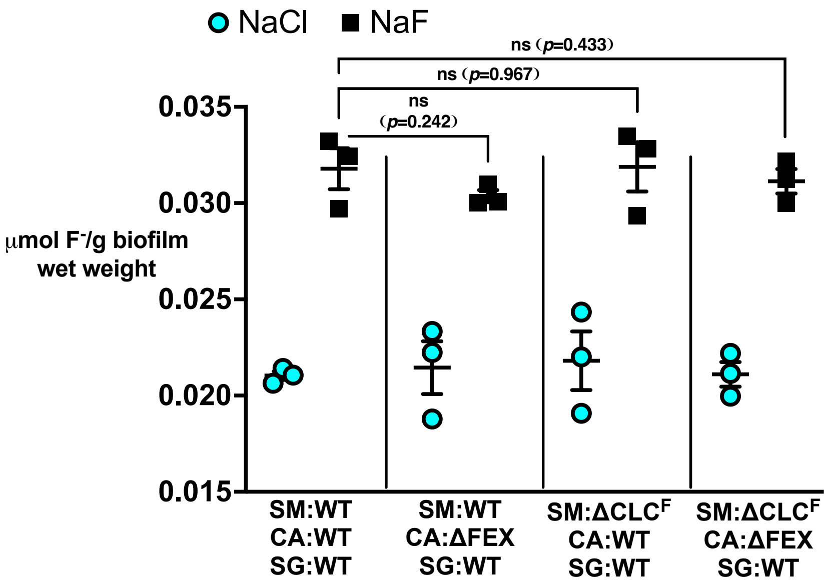

Supplementary Fig. 4. Fluoride content of biofilm combinations exposed to 0.3 mM NaCl or NaF at the end of the 5-day experiment. For all panels, datapoints represent mean and SEM of three independent experiments (*n*=3). Significance was calculated using two-way analysis of variance (ANOVA) followed by Fisher's LSD test and is represented as '*' ($p < 0.05$).

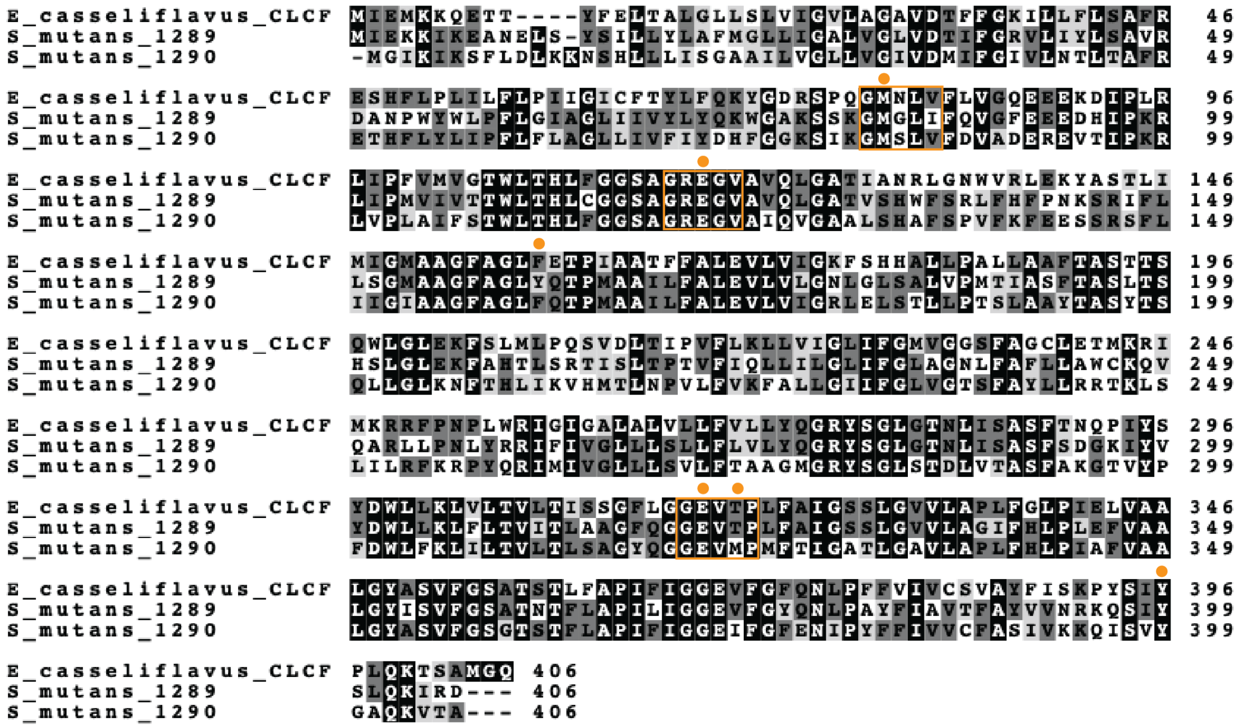

Supplementary Figure 5. Sequence alignment of *Enterococcus casseliflavus* CLC^F (GenBank EEV30821.1), *S. mutans* ORF 1289 (NCBI Protein ID: AAN58966), and *S. mutans* ORF 1290 (NCBI Protein ID: AAN58967.1). Shades of gray indicate the degree of conservation among the three sequences (black is completely conserved and white is not conserved). Motifs that distinguish fluoride transporters from chloride transporters are boxed in orange(22, 40). Residues that were observed to coordinate fluoride ion in the *E. casseliflavus* CLC^F structure(39) are indicated by orange dots.

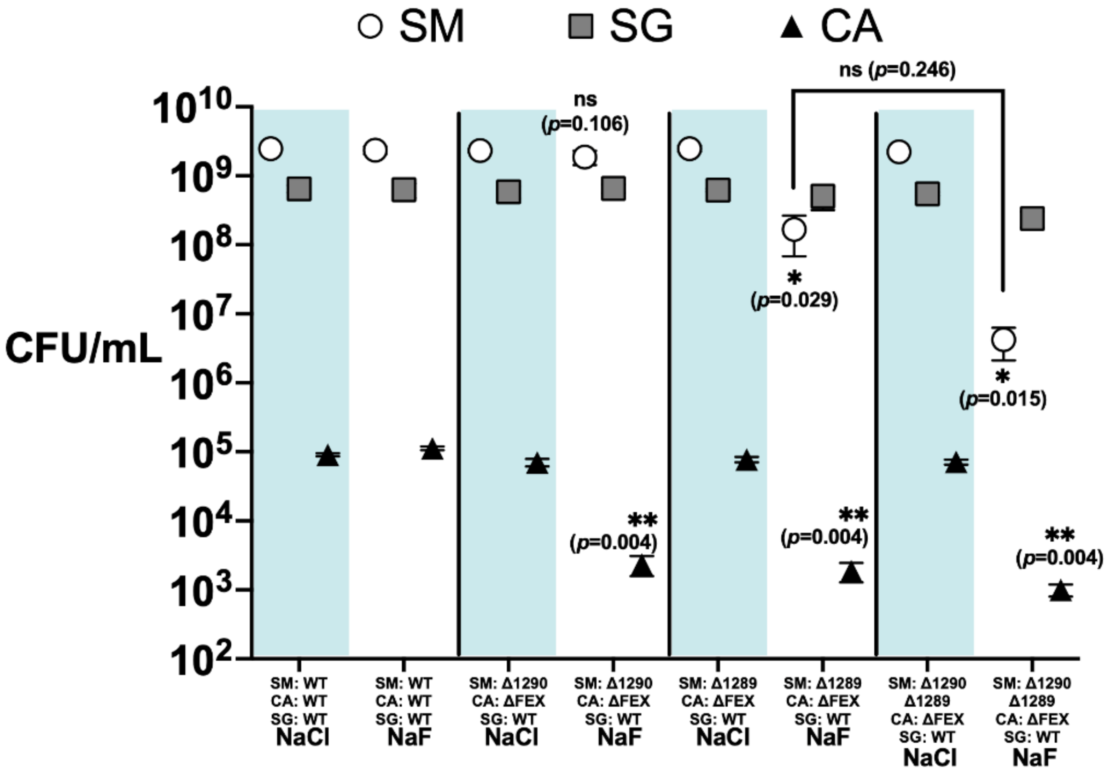

Supplementary Fig. 6. CFUs of *S. mutans*, *S. gordonii*, and *C. albicans* harvested from three-species (*S. mutans, S. gordonii, C. albicans*) biofilms derived from Δ1290 or Δ1289 or Δ1290Δ1289 *S. mutans*, ΔFEX *C. albicans* or the respective WT strains after 24 h growth in the presence of 0.2 mM NaCl or NaF. The datapoints represent mean and SEM of three independent experiments (*n*=3). Statistical significance relative to the all-WT biofilms is shown. Significance was calculated using two-way analysis of variance (ANOVA) followed by Fisher's LSD test. Statistical significance is represented as '*' ($p < 0.05$), '**' ($p < 0.01$) and '***' ($p < 0.001$).

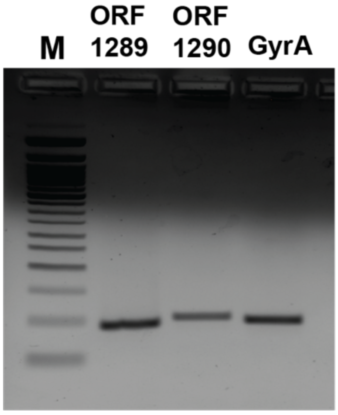

Supplementary Figure 7. Amplification of ORF 1289, ORF 1290, and reference gene GyrA by primers used for qPCR.

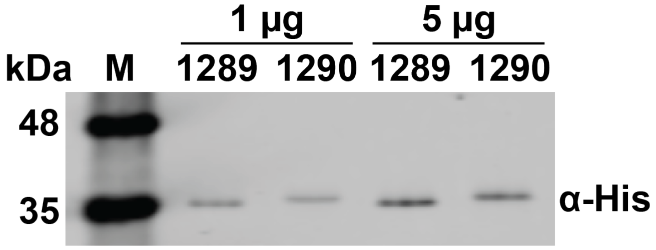

Supplementary Figure 8. Western blot of proteoliposomes reconstituted with 1289 or 1290, at 1 or 5 μg protein/mg lipid, as indicated. Protein detected with anti-His tag primary antibody (Genscript, Piscataway, NJ). The ratio of band intensities (1290/1289), measured using ImageJ(53), is 0.92 at 1 μg protein/mg lipid and 0.81 at 5 μg protein/mg lipid.

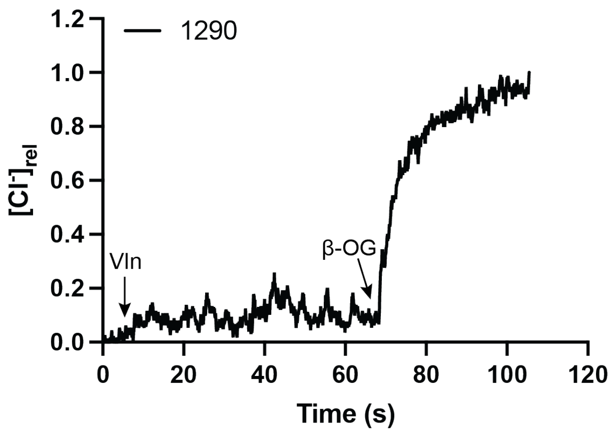

Supplementary Figure 9. Representative trace showing chloride efflux from proteoliposomes containing 1290. Protein-mediated chloride efflux is initiated by valinomycin addition (arrow). At the end of the experiment, proteoliposomes are solubilized by addition of detergent (arrow), releasing all encapsulated chloride. Trace is representative of three independent experiments.

Supplementary Table 1: List of primers used in the study.

Primer	Sequence
1290-up-f	ATGAAGAGAATACATGTAAGATATAAGCAAATTAG
1290-up-r	GAAAATCGATTTAATCCCCATCTAATGCTCTCCTT
1290-dw-f	CTTGCATATGTATCACCTGATTAATTAAAAAGAAG
1289-up-r	GTTTCCGCCAAATGCTTTCTTTTTATAAAATACGT
1289-dw-f	TCCAAGGAGCAGAAAAGTAGCTTACTCTTTTTGAG
1289-dw-r	ATGGAAAAAAGAAACGAAGATTATTCGGCATTATT
N-1290-up-f	GCAAATTAGACGCTTTTTTCTTTTACTGGGACTTG
N-1289-dw-r	AATTAGTTGTCGGTATCGGTGAGGTTTCTAAAATC
Spec^r-f	TGGGGATTAAATCGATTTTCGTTCGTGAATACATG
Spec^r-r	TCAGGTGATACATATGCAAGGGTTTATTGTTTTCT
Em^r-f	AGAAAGCATTTGGCGGAAACGTAAAAGAAGTTATG
Em^r-r	CTACTTTTCTGCTCCTTGGAAGCTGTCAGTAGTAT
Spec^{r}2-r	TTCCGCCAAATGCTTTCTTTTTATAAAATACGTGA
Em^{r}2-f	GAAAGCATTTGGCGGAAACGTAAAAGAAGTTATGG
1289-qPCR-f	GTGGAGAAGTGACGCCTTTAT
1289-qPCR-r	CCAGAGCTGCCACAAATTCTA
1290-qPCR-f	AAGGAGTGGCTATTCAGGTAGG
1290-qPCR-r	GCCAGCAGCGATTCCAATAA
gyrA-qPCR-f	ACAGGTGATGTCATGGGTAAAT
gyrA-qPCR-r	AACCAACATGTGACGGTAAGA

Supplementary Table 2: Statistical significance table for microbial quantification in polystyrene stalks. Significance was calculated using two-way analysis of variance (ANOVA) followed by Fisher's LSD test implemented in GraphPad Prism 8. Statistical significance is represented as '*' (p< 0.05), '**' (p< 0.01) and '***' (p< 0.001).

**Organism**	**Combination**	**Treatments**	**Summary**	**Adjusted '*p*' Value**
Microbial quantification of biofilm formed with single or both mutant strains of *S. mutans* or *C. albicans*				
S. mutans	WT (3 species)	NaCl vs. NaF	ns	0.8126
	SM (ΔCLC^F)	NaCl vs. NaF	*	0.0103
	CA (ΔFEX)	NaCl vs. NaF	**	0.0021
	SM (ΔCLC^F)- CA (ΔFEX)	NaCl vs. NaF	**	0.004
	WT (3 species) vs. SM (ΔCLC^F)	NaF	**	0.0016
	WT (3 species) vs. CA (ΔFEX)	NaF	**	0.0032
	WT (3 species) vs. SM (ΔCLC^F)- CA (ΔFEX)	NaF	**	0.0015
C. albicans	WT (3 species)	NaCl vs. NaF	ns	0.7473
	SM (ΔCLC^F)	NaCl vs. NaF	ns	0.0828
	CA (ΔFEX)	NaCl vs. NaF	*	0.0184
	SM (ΔCLC^F)- CA (ΔFEX)	NaCl vs. NaF	*	0.0167
	WT (3 species) vs. SM (ΔCLC^F)	NaF	*	0.0412
	WT (3 species) vs. CA (ΔFEX)	NaF	*	0.013
	WT (3 species) vs. SM (ΔCLC^F)-	NaF	**	0.0067

	CA (ΔFEX)			
S. gordonii	WT (3 species)	NaCl vs. NaF	ns	0.821
	SM (ΔCLC^F)	NaCl vs. NaF	ns	0.1212
	CA (ΔFEX)	NaCl vs. NaF	ns	0.0821
	SM (ΔCLC^F)-<br>CA (ΔFEX)	NaCl vs. NaF	ns	0.0671
	WT (3 species) vs.<br>SM (ΔCLC^F)	NaF	ns	0.182
	WT (3 species) vs.<br>CA (ΔFEX)	NaF	ns	0.3416
	WT (3 species) vs.<br>SM (ΔCLC^F)-<br>CA (ΔFEX)	NaF	ns	0.0767
Microbial quantification of biofilm formed with strains of *S. mutans* with either or both the CLC^F ORFs knocked out, *C. albicans* ΔFEX and *S. gordonii* WT				
S. mutans	WT (3 species)	NaCl vs. NaF	ns	0.7687
	SM (Δ1290)-<br>CA (ΔFEX)	NaCl vs. NaF	ns	0.4119
	SM (Δ1289)-<br>CA (ΔFEX)	NaCl vs. NaF	*	0.0211
	SM (Δ1290Δ1289)-<br>CA (ΔFEX)	NaCl vs. NaF	**	0.0019
	WT (3 species) vs.<br>SM (Δ1290)-<br>CA (ΔFEX)	NaF	ns	0.1060
	WT (3 species) vs.<br>SM (Δ1289)-<br>CA (ΔFEX)	NaF	*	0.0291
	WT (3 species) vs.<br>SM (Δ1290Δ1289)-	NaF	*	0.0151

	CA (ΔFEX)			
	SM (Δ1289)-<br>CA (ΔFEX) vs.<br>SM (Δ1290Δ1289)-<br>CA (ΔFEX)	NaF	ns	0.2460
C. albicans	WT (3 species)	NaCl vs. NaF	ns	0.0533
	SM (Δ1290)-<br>CA (ΔFEX)	NaCl vs. NaF	*	0.0178
	SM (Δ1289)-<br>CA (ΔFEX)	NaCl vs. NaF	*	0.0100
	SM (Δ1290Δ1289)-<br>CA (ΔFEX)	NaCl vs. NaF	**	0.0066
	WT (3 species) vs.<br>SM (Δ1290)-<br>CA (ΔFEX)	NaF	**	0.0037
	WT (3 species) vs.<br>SM (Δ1289)-<br>CA (ΔFEX)	NaF	**	0.0037
	WT (3 species) vs.<br>SM (Δ1290Δ1289)-<br>CA (ΔFEX)	NaF	**	0.0040
S. gordonii	WT (3 species)	NaCl vs. NaF	ns	0.8277
	SM (Δ1290)-<br>CA (ΔFEX)	NaCl vs. NaF	ns	0.7203
	SM (Δ1289)-<br>CA (ΔFEX)	NaCl vs. NaF	ns	0.6461
	SM (Δ1290Δ1289)-<br>CA (ΔFEX)	NaCl vs. NaF	*	0.0237
	WT (3 species) vs.<br>SM (Δ1290)-	NaF	ns	0.8297

	CA (ΔFEX)			
	WT (3 species) vs. SM (Δ1289)-CA (ΔFEX)	NaF	ns	0.2863
	WT (3 species) vs. SM (Δ1290Δ1289)-CA (ΔFEX)	NaF	ns	0.2063

Supplementary Table 3: Statistical significance table for HA disc experiments. Significance was calculated using two-way analysis of variance (ANOVA) followed by Fisher's LSD test implemented in GraphPad Prism 8. Statistical significance is represented as '*' ($p< 0.05$), '**' ($p< 0.01$) and '***' ($p< 0.001$).

**Timeline**	**Combinations**	**Treatments**	**Summary**	**Adjusted '*p*' Value**
**Temporal changes of pH in media**				
D1-PM	WT (3 species)	NaCl vs. NaF (no treatment)	ns	0.5286
	SM (ΔCLC^F)	NaCl vs. NaF (no treatment)	ns	0.4226
	CA (ΔFEX)	NaCl vs. NaF (no treatment)	ns	0.8259
	SM (ΔCLC^F)- CA (ΔFEX)	NaCl vs. NaF (no treatment)	ns	0.6784
	WT (3 species) vs. SM (ΔCLC^F)	NaF (no treatment)	ns	0.4226
	WT (3 species) vs. CA (ΔFEX)	NaF (no treatment)	ns	0.5200
	WT (3 species) vs. SM (ΔCLC^F)- CA (ΔFEX)	NaF (no treatment)	ns	0.6349
D2-AM	WT (3 species)	NaCl vs. NaF	*	0.0377
	SM (ΔCLC^F)	NaCl vs. NaF	**	0.0098
	CA (ΔFEX)	NaCl vs. NaF	**	0.0047
	SM (ΔCLC^F)- CA (ΔFEX)	NaCl vs. NaF	*	0.0229
	WT (3 species) vs. SM (ΔCLC^F)	NaF	*	0.027
	WT (3 species) vs. CA (ΔFEX)	NaF	ns	0.4639

	WT (3 species) vs. SM (ΔCLC^F)-CA (ΔFEX)	NaF	*	0.0198
D2-PM	WT (3 species)	NaCl vs. NaF	ns	0.0551
	SM (ΔCLC^F)	NaCl vs. NaF	*	0.0446
	CA (ΔFEX)	NaCl vs. NaF	ns	0.0562
	SM (ΔCLC^F)-CA (ΔFEX)	NaCl vs. NaF	ns	0.0824
	WT (3 species) vs. SM (ΔCLC^F)	NaF	ns	0.0598
	WT (3 species) vs. CA (ΔFEX)	NaF	ns	0.1329
	WT (3 species) vs. SM (ΔCLC^F)-CA (ΔFEX)	NaF	ns	0.1013
D3-AM	WT (3 species)	NaCl vs. NaF	ns	0.149
	SM (ΔCLC^F)	NaCl vs. NaF	ns	0.0806
	CA (ΔFEX)	NaCl vs. NaF	*	0.0351
	SM (ΔCLC^F)-CA (ΔFEX)	NaCl vs. NaF	ns	0.0858
	WT (3 species) vs. SM (ΔCLC^F)	NaF	ns	0.0977
	WT (3 species) vs. CA (ΔFEX)	NaF	ns	0.1221
	WT (3 species) vs. SM (ΔCLC^F)-CA (ΔFEX)	NaF	ns	0.0885
D3-PM	WT (3 species)	NaCl vs. NaF	*	0.0311
	SM (ΔCLC^F)	NaCl vs. NaF	**	0.0054
	CA (ΔFEX)	NaCl vs. NaF	*	0.0297

	SM (ΔCLC^F)-CA (ΔFEX)	NaCl vs. NaF	**	0.0095
	WT (3 species) vs. SM (ΔCLC^F)	NaF	**	0.0089
	WT (3 species) vs. CA (ΔFEX)	NaF	**	0.0059
	WT (3 species) vs. SM (ΔCLC^F)-CA (ΔFEX)	NaF	**	0.0034
D4-AM	WT (3 species)	NaCl vs. NaF	ns	0.2029
	SM (ΔCLC^F)	NaCl vs. NaF	ns	0.0927
	CA (ΔFEX)	NaCl vs. NaF	*	0.0258
	SM (ΔCLC^F)-CA (ΔFEX)	NaCl vs. NaF	ns	0.1071
	WT (3 species) vs. SM (ΔCLC^F)	NaF	ns	0.0728
	WT (3 species) vs. CA (ΔFEX)	NaF	ns	0.3715
	WT (3 species) vs. SM (ΔCLC^F)-CA (ΔFEX)	NaF	ns	0.1235
D4-PM	WT (3 species)	NaCl vs. NaF	ns	0.0615
	SM (ΔCLC^F)	NaCl vs. NaF	ns	0.0513
	CA (ΔFEX)	NaCl vs. NaF	ns	0.0598
	SM (ΔCLC^F)-CA (ΔFEX)	NaCl vs. NaF	ns	0.0539
	WT (3 species) vs. SM (ΔCLC^F)	NaF	ns	0.1183
	WT (3 species) vs. CA (ΔFEX)	NaF	ns	0.1801

	WT (3 species) vs. SM (ΔCLC^F)-CA (ΔFEX)	NaF	ns	0.0803
D5-AM	WT (3 species)	NaCl vs. NaF	ns	0.5073
	SM (ΔCLC^F)	NaCl vs. NaF	ns	0.1593
	CA (ΔFEX)	NaCl vs. NaF	ns	0.2841
	SM (ΔCLC^F)-CA (ΔFEX)	NaCl vs. NaF	ns	0.1801
	WT (3 species) vs. SM (ΔCLC^F)	NaF	*	0.0373
	WT (3 species) vs. CA (ΔFEX)	NaF	ns	0.3235
	WT (3 species) vs. SM (ΔCLC^F)-CA (ΔFEX)	NaF	ns	0.0925
**Temporal changes in calcium release in media**				
D1-PM	WT (3 species)	NaCl vs. NaF (no treatment)	ns	0.4226
	SM (ΔCLC^F)	NaCl vs. NaF (no treatment)	ns	0.4778
	CA (ΔFEX)	NaCl vs. NaF (no treatment)	ns	>0.9999
	SM (ΔCLC^F)-CA (ΔFEX)	NaCl vs. NaF (no treatment)	ns	0.7072
	WT (3 species) vs. SM (ΔCLC^F)	NaF (no treatment)	ns	0.7735
	WT (3 species) vs. CA (ΔFEX)	NaF (no treatment)	ns	0.4226
	WT (3 species) vs.	NaF	ns	0.6784

	SM (ΔCLC^F)-<br>CA (ΔFEX)	(no treatment)		
D2-AM	WT (3 species)	NaCl vs. NaF	ns	0.0572
	SM (ΔCLC^F)	NaCl vs. NaF	ns	0.1576
	CA (ΔFEX)	NaCl vs. NaF	ns	0.0797
	SM (ΔCLC^F)-<br>CA (ΔFEX)	NaCl vs. NaF	**	0.0025
	WT (3 species) vs.<br>SM (ΔCLC^F)	NaF	ns	0.4226
	WT (3 species) vs.<br>CA (ΔFEX)	NaF	*	0.0377
	WT (3 species) vs.<br>SM (ΔCLC^F)-<br>CA (ΔFEX)	NaF	**	0.0015
D2-PM	WT (3 species)	NaCl vs. NaF	ns	0.1835
	SM (ΔCLC^F)	NaCl vs. NaF	*	0.0321
	CA (ΔFEX)	NaCl vs. NaF	ns	0.1047
	SM (ΔCLC^F)-<br>CA (ΔFEX)	NaCl vs. NaF	**	0.0035
	WT (3 species) vs.<br>SM (ΔCLC^F)	NaF	*	0.0124
	WT (3 species) vs.<br>CA (ΔFEX)	NaF	*	0.0198
	WT (3 species) vs.<br>SM (ΔCLC^F)-<br>CA (ΔFEX)	NaF	**	0.0032
D3-AM	WT (3 species)	NaCl vs. NaF	*	0.0289
	SM (ΔCLC^F)	NaCl vs. NaF	*	0.0224
	CA (ΔFEX)	NaCl vs. NaF	*	0.0279
	SM (ΔCLC^F)-	NaCl vs. NaF	**	0.0039

	CA (ΔFEX)			
	WT (3 species) vs. SM (ΔCLC^F)	NaF	**	0.0075
	WT (3 species) vs. CA (ΔFEX)	NaF	*	0.0377
	WT (3 species) vs. SM (ΔCLC^F)-CA (ΔFEX)	NaF	**	0.0059
D3-PM	WT (3 species)	NaCl vs. NaF	**	0.0038
	SM (ΔCLC^F)	NaCl vs. NaF	*	0.0126
	CA (ΔFEX)	NaCl vs. NaF	*	0.0108
	SM (ΔCLC^F)-CA (ΔFEX)	NaCl vs. NaF	***	0.0005
	WT (3 species) vs. SM (ΔCLC^F)	NaF	***	0.0004
	WT (3 species) vs. CA (ΔFEX)	NaF	*	0.0151
	WT (3 species) vs. SM (ΔCLC^F)-CA (ΔFEX)	NaF	**	0.004
D4-AM	WT (3 species)	NaCl vs. NaF	*	0.0371
	SM (ΔCLC^F)	NaCl vs. NaF	*	0.0342
	CA (ΔFEX)	NaCl vs. NaF	**	0.0059
	SM (ΔCLC^F)-CA (ΔFEX)	NaCl vs. NaF	*	0.0102
	WT (3 species) vs. SM (ΔCLC^F)	NaF	**	0.0091
	WT (3 species) vs. CA (ΔFEX)	NaF	**	0.0099
	WT (3 species) vs.	NaF	**	0.0014

	SM (ΔCLC^{F})- CA (ΔFEX)			
D4-PM	WT (3 species)	NaCl vs. NaF	ns	0.0822
	SM (ΔCLC^{F})	NaCl vs. NaF	*	0.0392
	CA (ΔFEX)	NaCl vs. NaF	**	0.0028
	SM (ΔCLC^{F})- CA (ΔFEX)	NaCl vs. NaF	**	0.0022
	WT (3 species) vs. SM (ΔCLC^{F})	NaF	ns	0.0583
	WT (3 species) vs. CA (ΔFEX)	NaF	ns	0.0902
	WT (3 species) vs. SM (ΔCLC^{F})- CA (ΔFEX)	NaF	**	0.0088
D5-AM	WT (3 species)	NaCl vs. NaF	*	0.0491
	SM (ΔCLC^{F})	NaCl vs. NaF	ns	0.0759
	CA (ΔFEX)	NaCl vs. NaF	ns	0.1239
	SM (ΔCLC^{F})- CA (ΔFEX)	NaCl vs. NaF	*	0.0413
	WT (3 species) vs. SM (ΔCLC^{F})	NaF	ns	0.1734
	WT (3 species) vs. CA (ΔFEX)	NaF	ns	0.3169
	WT (3 species) vs. SM (ΔCLC^{F})- CA (ΔFEX)	NaF	*	0.0143
**Microbial quantification**				
**Organism**	**Combinations**	**Treatments**	**Summary**	**Adjusted '*p*' Value**

S. mutans	WT (3 species)	NaCl vs. NaF	ns	0.497
	SM (ΔCLC^F)	NaCl vs. NaF	*	0.0101
	CA (ΔFEX)	NaCl vs. NaF	ns	0.907
	SM (ΔCLC^F)-CA (ΔFEX)	NaCl vs. NaF	**	0.0057
	WT (3 species) vs. SM (ΔCLC^F)	NaF	*	0.0401
	WT (3 species) vs. CA (ΔFEX)	NaF	ns	0.5959
	WT (3 species) vs. SM (ΔCLC^F)-CA (ΔFEX)	NaF	*	0.0102
S. gordonii	WT (3 species)	NaCl vs. NaF	ns	0.8209
	SM (ΔCLC^F)	NaCl vs. NaF	ns	0.0632
	CA (ΔFEX)	NaCl vs. NaF	ns	0.9768
	SM (ΔCLC^F)-CA (ΔFEX)	NaCl vs. NaF	*	0.0238
	WT (3 species) vs. SM (ΔCLC^F)	NaF	ns	0.0575
	WT (3 species) vs. CA (ΔFEX)	NaF	ns	0.8031
	WT (3 species) vs. SM (ΔCLC^F)-CA (ΔFEX)	NaF	**	0.0026
C. albicans	WT (3 species)	NaCl vs. NaF	ns	0.1473
	SM (ΔCLC^F)	NaCl vs. NaF	ns	0.2057
	CA (ΔFEX)	NaCl vs. NaF	**	0.0047
	SM (ΔCLC^F)-CA (ΔFEX)	NaCl vs. NaF	**	0.0022
	WT (3 species) vs.	NaF	ns	0.1383

	SM (ΔCLC^F)			
	WT (3 species) vs. CA (ΔFEX)	NaF	***	0.0009
	WT (3 species) vs. SM (ΔCLC^F)-CA (ΔFEX)	NaF	***	0.0009
**Temporal microbial quantification among NaF-treatments: *S. mutans***				
**Timeline**	**Combinations**	**Treatments**	**Summary**	**Adjusted ‘*p*’ Value**
D1-PM	WT (3 species) vs. CA (ΔFEX)	No treatment	ns	0.579
	WT (3 species) vs. SM (ΔCLC^F)	No treatment	ns	0.4631
	WT (3 species) vs. SM (ΔCLC^F)-CA (ΔFEX)	No treatment	ns	0.3802
D2-AM	WT (3 species) vs. CA (ΔFEX)	NaF	ns	0.225
	WT (3 species) vs. SM (ΔCLC^F)	NaF	ns	0.6914
	WT (3 species) vs. SM (ΔCLC^F)-CA (ΔFEX)	NaF	*	0.011
D2-PM	WT (3 species) vs. CA (ΔFEX)	NaF	ns	0.464
	WT (3 species) vs. SM (ΔCLC^F)	NaF	ns	0.0596
	WT (3 species) vs. SM (ΔCLC^F)-	NaF	**	0.0055

	CA (ΔFEX)			
D3-AM	WT (3 species) vs. CA (ΔFEX)	NaF	ns	0.528
	WT (3 species) vs. SM (ΔCLC^F)	NaF	**	0.0017
	WT (3 species) vs. SM (ΔCLC^F)-CA (ΔFEX)	NaF	**	0.0034
D3-PM	WT (3 species) vs. CA (ΔFEX)	NaF	ns	0.551
	WT (3 species) vs. SM (ΔCLC^F)	NaF	**	0.0032
	WT (3 species) vs. SM (ΔCLC^F)-CA (ΔFEX)	NaF	**	0.0024
D4-AM	WT (3 species) vs. CA (ΔFEX)	NaF	ns	0.157
	WT (3 species) vs. SM (ΔCLC^F)	NaF	**	0.0058
	WT (3 species) vs. SM (ΔCLC^F)-CA (ΔFEX)	NaF	***	0.0009
D4-PM	WT (3 species) vs. CA (ΔFEX)	NaF	ns	0.214
	WT (3 species) vs. SM (ΔCLC^F)	NaF	**	0.0038
	WT (3 species) vs. SM (ΔCLC^F)-CA (ΔFEX)	NaF	**	0.0019
D5-AM	WT (3 species) vs.	NaF	ns	0.257

	CA (ΔFEX)			
	WT (3 species) vs. SM (ΔCLC^F)	NaF	**	0.0048
	WT (3 species) vs. SM (ΔCLC^F)- CA (ΔFEX)	NaF	**	0.0011
**Temporal microbial quantification among NaF-treatments: *C. albicans***				
D1-PM	WT (3 species) vs. CA (ΔFEX)	No treatment	ns	0.7650
	WT (3 species) vs. SM (ΔCLC^F)	No treatment	ns	0.551
	WT (3 species) vs. SM (ΔCLC^F)- CA (ΔFEX)	No treatment	ns	0.6784
D2-AM	WT (3 species) vs. CA (ΔFEX)	NaF	ns	0.6242
	WT (3 species) vs. SM (ΔCLC^F)	NaF	**	0.008
	WT (3 species) vs. SM (ΔCLC^F)- CA (ΔFEX)	NaF	ns	0.0742
D2-PM	WT (3 species) vs. CA (ΔFEX)	NaF	ns	0.0667
	WT (3 species) vs. SM (ΔCLC^F)	NaF	ns	0.130
	WT (3 species) vs. SM (ΔCLC^F)- CA (ΔFEX)	NaF	**	0.0083
D3-AM	WT (3 species) vs.	NaF	*	0.0111

	CA (ΔFEX)			
	WT (3 species) vs. SM (ΔCLC^{F})	NaF	ns	0.078
	WT (3 species) vs. SM (ΔCLC^{F})-CA (ΔFEX)	NaF	**	0.0047
D3-PM	WT (3 species) vs. CA (ΔFEX)	NaF	**	0.0013
	WT (3 species) vs. SM (ΔCLC^{F})	NaF	*	0.031
	WT (3 species) vs. SM (ΔCLC^{F})-CA (ΔFEX)	NaF	**	0.004
D4-AM	WT (3 species) vs. CA (ΔFEX)	NaF	**	0.0068
	WT (3 species) vs. SM (ΔCLC^{F})	NaF	ns	0.132
	WT (3 species) vs. SM (ΔCLC^{F})-CA (ΔFEX)	NaF	*	0.0126
D4-PM	WT (3 species) vs. CA (ΔFEX)	NaF	**	0.0039
	WT (3 species) vs. SM (ΔCLC^{F})	NaF	ns	0.211
	WT (3 species) vs. SM (ΔCLC^{F})-CA (ΔFEX)	NaF	**	0.007
D5-AM	WT (3 species) vs. CA (ΔFEX)	NaF	***	0.0008
	WT (3 species) vs.	NaF	ns	0.221

	SM (ΔCLC^F)			
	WT (3 species) vs. SM (ΔCLC^F)-CA (ΔFEX)	NaF	**	0.0051
**Temporal microbial quantification among NaF-treatments: *S. gordonii***				
D1-PM	WT (3 species) vs. SM (ΔCLC^F)	No treatment	ns	0.7418
	WT (3 species) vs. CA (ΔFEX)	No treatment	ns	0.2254
	WT (3 species) vs. SM (ΔCLC^F)-CA (ΔFEX)	No treatment	ns	0.6667
D2-AM	WT (3 species) vs. SM (ΔCLC^F)	NaF	ns	0.4226
	WT (3 species) vs. CA (ΔFEX)	NaF	*	0.0377
	WT (3 species) vs. SM (ΔCLC^F)-CA (ΔFEX)	NaF	ns	>0.9999
D2-PM	WT (3 species) vs. SM (ΔCLC^F)	NaF	ns	0.0848
	WT (3 species) vs. CA (ΔFEX)	NaF	*	0.0202
	WT (3 species) vs. SM (ΔCLC^F)-CA (ΔFEX)	NaF	*	0.0109
D3-AM	WT (3 species) vs. SM (ΔCLC^F)	NaF	**	0.0034
	WT (3 species) vs.	NaF	*	0.0339

	CA (ΔFEX)			
	WT (3 species) vs. SM (ΔCLC^{F})-CA (ΔFEX)	NaF	**	0.0019
D3-PM	WT (3 species) vs. SM (ΔCLC^{F})	NaF	ns	0.0942
	WT (3 species) vs. CA (ΔFEX)	NaF	*	0.0153
	WT (3 species) vs. SM (ΔCLC^{F})-CA (ΔFEX)	NaF	*	0.0136
D4-AM	WT (3 species) vs. SM (ΔCLC^{F})	NaF	*	0.0377
	WT (3 species) vs. CA (ΔFEX)	NaF	ns	0.1086
	WT (3 species) vs. SM (ΔCLC^{F})-CA (ΔFEX)	NaF	*	0.0351
D4-PM	WT (3 species) vs. SM (ΔCLC^{F})	NaF	*	0.0377
	WT (3 species) vs. CA (ΔFEX)	NaF	ns	0.6349
	WT (3 species) vs. SM (ΔCLC^{F})-CA (ΔFEX)	NaF	*	0.0202
D5-AM	WT (3 species) vs. SM (ΔCLC^{F})	NaF	*	0.0377
	WT (3 species) vs. CA (ΔFEX)	NaF	ns	0.1994
	WT (3 species) vs.	NaF	**	0.0091

	SM (ΔCLC^F)-<br>CA (ΔFEX)			
